# Use of Mobile Phones and Radiofrequency-Emitting Devices in the COSMOS-France Cohort

**DOI:** 10.3390/ijerph21111514

**Published:** 2024-11-14

**Authors:** Isabelle Deltour, Florence Guida, Céline Ribet, Marie Zins, Marcel Goldberg, Joachim Schüz

**Affiliations:** 1 Environment and Lifestyle Epidemiology Branch, International Agency for Research on Cancer (IARC/WHO), 69366 Lyon, France; guidaf@iarc.who.int (F.G.); schuzj@iarc.who.int (J.S.); 2 Université Paris Cité, UVSQ, INSERM UMS 011, 94800 Villejuif, France; celine.ribet@inserm.fr (C.R.); marie.zins@inserm.fr (M.Z.); marcel.goldberg@inserm.fr (M.G.)

**Keywords:** cohort study, electromagnetic fields, non-ionizing radiation, cordless phone, Wi-Fi, questionnaire, lifestyle exposure

## Abstract

COSMOS-France is the French part of the COSMOS project, an international prospective cohort study that investigates whether the use of mobile phones and other wireless technologies is associated with health effects and symptoms (cancers, cardiovascular diseases, neurologic pathologies, tinnitus, headaches, or sleep and mood disturbances). Here, we provide the first descriptive results of COSMOS-France, a cohort nested in the general population-based cohort of adults named Constances. Methods: A total of 39,284 Constances volunteers were invited to participate in the COSMOS-France study during the pilot (2017) and main recruitment phase (2019). Participants were asked to complete detailed questionnaires on their mobile phone use, health conditions, and personal characteristics. We examined the association between mobile phone use, including usage for calls and Voice over Internet Protocol (VoIP), cordless phone use, and Wi-Fi usage with age, sex, education, smoking status, body mass index (BMI), and handedness. Results: The participation rate was 48.4%, resulting in 18,502 questionnaires in the analyzed dataset. Mobile phone use was reported by 96.1% (N = 17,782). Users reported typically calling 5–29 min per week (37.1%, N = 6600), making one to four calls per day (52.9%, N = 9408), using one phone (83.9%, N = 14,921) and not sharing it (80.4% N = 14,295), mostly using the phone on the side of the head of their dominant hand (59.1%, N = 10,300), not using loudspeakers or hands-free kits, and not using VoIP (84.9% N = 15,088). Individuals’ age and sex modified this picture, sometimes markedly. Education and smoking status were associated with ever use and call duration, but neither BMI nor handedness was. Cordless phone use was reported by 66.0% of the population, and Wi-Fi use was reported by 88.4%. Conclusion: In this cross-sectional presentation of contemporary mobile phone usage in France, age and sex were important determinants of use patterns.

## 1. Introduction

COSMOS is an international prospective cohort study designed to investigate whether the use of mobile phones and other wireless technologies is associated with adverse health effects (focussing on cancers, cerebro- and cardiovascular diseases, neurological disorders and motor neuron diseases, tinnitus, headaches, and sleep and mood disturbances) [1]. The acronym stands for COhort Study of MObile phone uSers and health. The international cohort currently consists of six cohorts, followed up prospectively, with COSMOS-France being the most recent addition to the project [1,2,3,4]. This large multinational cohort has been gradually established: participant recruitment occurred in 2007 and 2009 in Denmark, 2008 and 2009 in Sweden, 2009 and 2011 in Finland, in multiple waves between 2009 and 2012 in the United Kingdom, in 2011–2012 in the Netherlands, and in 2017 and 2019 in France [1,2,3]. In all COSMOS components, participants were adults aged 18 years or older at recruitment, though the upper age limit varied: the maximum age at recruitment was 65 years in the Swedish COSMOS cohort, while there was no age limit in the COSMOS-UK cohort.

Validation studies comparing self-reported mobile phone usage with telephone traffic records have consistently identified significant random and systematic errors in self-reported usage, with discrepancies differing between cases and controls [5]. Case–control studies are therefore susceptible to recall bias, which complicates the interpretation of their results [6,7,8,9]. In contrast, a cohort study that prospectively collects detailed self-reported mobile phone use data can better clarify health relationships while allowing flexibility to investigate emerging technologies, such as 5G and Voice over Internet Protocol (VoIP) calls—facilitated by apps like Skype, WhatsApp, and Viber, which use the Internet to transfer voice data—and new usage patterns such as holding the phone away from the head while calling. Questionnaires remain the only feasible option for gathering certain individual usage patterns on a large scale, whereas the scientific value of collecting objective telephone traffic records has already been well demonstrated in the COSMOS analyses of sleep and brain tumour risks [4,10,11]. As per the COSMOS protocol, COSMOS-France participants were therefore asked to consent to their mobile phone traffic records being provided by their operators.

Few studies have specifically focused on describing patterns of radiofrequency- (RF) emitting equipment use in the general population, with some exceptions in studies on adolescents and young adults [12,13,14]. Descriptive tables of epidemiological studies that investigate associations between wireless phone use and health outcomes generally provide some information on usage, albeit with a different focus.

Sociodemographic characteristics such as age, sex, and education, smoking status, and body mass index (BMI) are known to be associated with many health outcomes and may act as confounder in the exposure–disease relationship if they are also associated with exposure. We also considered handedness to assess whether phone usage patterns differed among left-handed, ambidextrous, and right-handed individuals. Given the highly localized RF exposure when a phone is held to the head, some epidemiological risk analyses have examined the correlation between outcome laterality (often intracranial tumours) and exposure laterality. Handedness has also been associated with glioma risk in one study [15].

In this manuscript, we present the COSMOS-France cohort profile with respect to sociodemographic characteristics, smoking, BMI, and handedness and we present the first analyses of the self-reported use of RF-emitting devices, namely, mobile phones, cordless phones, and devices using Wi-Fi networks.

## 2. Methods

In France, participants of COSMOS were recruited from within the French general population cohort “Constances” (for details see doi.org/10.13143/inserm_constances [16]). Constances enrolled randomly selected adults aged 18 to 69 years during recruitment between 2012 and 2019 from among those registered with French national health insurance (*Sécurité Sociale*) and living in one of the twenty French departments (*départements*) covered by Constances in metropolitan France. Great efforts were made to achieve a representative sample for France in terms of age, sex, and socio-economic status. The Constances cohort includes 219,144 volunteers, whose health is followed through regular comprehensive health examinations at a Health Screening Centre, periodically repeated questionnaires, and record linkage with various French national administrative databases, without any time limit.

Constances volunteers formed the pool of individuals from which potential Cosmos participants were selected. A total of 41,000 Constances volunteers were invited to also participate in the COSMOS-France study: 1000 during the pilot phase of the study in December 2017, and 40,000 in January 2019 during the main recruitment phase. The random selection criteria for these Constances volunteers are detailed in Supplementary Material S1. All were sent a letter explaining the purpose of the study, an information leaflet, a consent form, and a pre-paid envelope to return the consent form; 30,500 individuals were sent a paper questionnaire, as this was indicated as their preferred option to respond to questionnaires for Constances, and 10,500 individuals were invited to fill in the COSMOS-France questionnaire via Constances’ internet portal. One month later, a reminder letter was sent to those who had not yet returned all their documents, and another month later, a second reminder letter, together with either a consent form or a paper copy of the questionnaire, and the respective pre-paid return envelope was sent to all those who had returned only one of the documents to date. On the consent form, participants had to indicate whether they allowed access to their telephone traffic data through mobile phone operators.

The paper and online questionnaire contained identical detailed questions on current and past mobile phone use and other characteristics of using mobile phones, such as, during calls, the preferred side of the head or the time spent not holding the phone to the ear; the usage of cordless phones; wireless network (Wi-Fi) usage while using a fixed personal computer, laptop, tablet, e-book reader, media player, or video game console; headaches, tinnitus, and sleep disturbances; and dominant handedness (right-handed, left-handed, or ambidextrous). The phrasing of the questions and the response options were the same as those used in the other countries’ COSMOS cohorts (see Supplementary Material S2: COSMOS-France questionnaire). Sex, month and year of birth, recruitment department (considered as a proxy for department of residence), attained number of years of schooling, tobacco smoking status (never smoked, former smoker, or current smoker), height, and weight were taken from the Constances database. Age at recruitment was calculated based on the date on which the COSMOS-France study questionnaires were sent. BMI was computed as weight divided by height squared, with weight expressed in kg and height in m, and participants were classified as underweight (<18.5 kg/m^2^), a healthy weight (18.5–24.9 kg/m^2^), overweight (25–29.9 kg/m^2^), or obese (≥30 kg/m^2^).

All analyses were carried out using Stata version 17. Missing data are shown as a separate category in the tables. Tests of significance and of trends were produced using logistic, polytomous (mlogit), ordered logistic (ologit), or generalized ordered logistic (gologit2) regressions, and linear mixed models were used to model longitudinal data, excluding missing values. Some of the written content was reviewed and polished by ChatGPT V4.0 to improve clarity, conciseness, and flow. Sentence structure, word choice, and grammar were refined without altering the substantive content or intended meaning of the original text.

The COSMOS-France study was approved by the IARC Ethics committee (IEC project 14–24), the French CCTIRS (15.024), the French Data Protection Authority (*Commission Nationale de l’Informatique et des Libertés*, CNIL) (authorization 915635), and the Constances scientific committee in 2014 and 2018. It is currently following the French CNIL’s MR-004 framework.

## 3. Results

**Figure 1:**
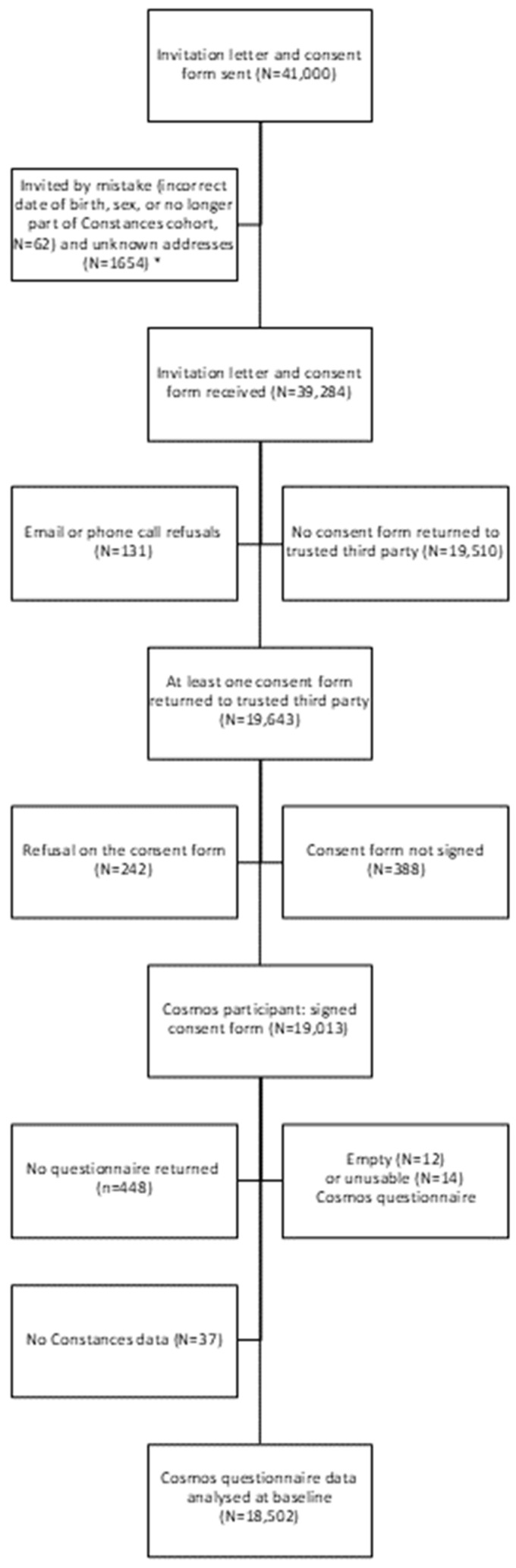
Flow chart for participation in the COSMOS-France study. Note: * excluded from the calculation of the participation rate.

Out of the 41,000 attempted contacts, 1716 invitation letters were sent by mistake (e.g., wrong or outdated postal address), 19,510 individuals did not return a consent form, 373 refused to participate in the study, and 388 returned but did not sign the consent form, leaving 19,013 individuals who returned a signed consent form. Of these, 18,502 had filled in the COSMOS-France questionnaire in such a way that the data could be used for this analysis (Figure 1). The participation rate was 48.4% (19,013 returning the signed consent form divided by 39,284 receiving the invitation letter) (Supplementary Table S1). The response rate was higher among older age groups. The proportion of participants who agreed to their operator data being accessed was 83.4% and was higher among younger participants. Almost all those who agreed to participate also returned a usable questionnaire.

The majority of participants was 60 years or older (N = 7415, 40.1%), while the proportion of participants below 30 years of age was small (N = 773, 4.2%) and comprised mostly women (55.5%, N = 10,262) (Table 1). The mean delay between recruitment in Constances and recruitment in COSMOS-France was 4.4 years (interquartile range, 3.5–5.3 years). Participants with 14 to 15 years of education formed the largest group (N = 4826, 26.1%). Younger people had had more years of education, except for those younger than 30 years old, an age group which could include participants who had not yet completed their education when they were recruited in Constances (Supplementary Figure S1). Comparing within the same age group, there were proportionally more women in the middle categories of education (between 12 and 16 years of education) and more men in the two extreme categories (either up to 11 years of education or 17 years of education or more). Overall, slightly less than half of the participants (N = 8828, 47.7%) reported that they had never smoked; smoking status varied substantially by age and sex group (Supplementary Figure S2). Overall, over half of the participants had a healthy weight (N = 10,183, 55,0%); this proportion was strongly influenced by age and sex (Supplementary Figure S3). Nearly nine in ten participants (N = 16,087, 87.0%) were right-handed; older participants reported being right-handed slightly more often than younger ones, while women reported being right-handed slightly more often than men (Supplementary Figure S4).

### 3.1. Use of Mobile Phones

Almost all participants reported using a mobile phone for voice calls (N = 17,782, 96.1%) during the 3 months before completing the questionnaire. The frequency of non-users (N = 692, 3.7%) increased with increasing age (p-trend < 0.01, not shown in table) and no use was more common among men than among women (Odds-Ratio (OR) = 1.18, 95% Confidence Interval (CI) 1.02–1.38) (Table 2). After adjusting for age and sex, people with the lowest education were more likely to be non-users (p-trend < 0.01); current and former smokers were less likely to be non-users than those who had never smoked (education-adjusted-OR for those who had ever smoked = 0.62 CI 0.47–0.81). BMI and left-handedness were not associated with the non-use of mobile phone (*p*-value = 0.31 and 0.30) (Supplementary Table S2).

There were no uniform patterns in the duration of calls with either age or sex (Table 2, see also Supplementary Figure S5). Among mobile phone users, 37.1% of them (N = 6600) reported calling for between 5 and 29 min per week. Older women reported shorter call durations than younger women. For example, 48.2% of women aged 60 and above reported calling for less than 30 min per week, compared to 28.9% of women under 30 years old. In contrast, men aged 30–59 years reported calling for over 4 h per week more frequently than other age groups. For example, 17.3% of men aged 30–44 and 12.8% of men aged 45–59 reported calling for over 4 h per week, compared to 7.7% of men under 30 years old. The men above 60 years old declared the shortest call durations of all sex and age groups. After adjusting for age and sex, people with longer education periods reported calling longer durations than those with shorter education periods (p-trend < 0.01) (Supplementary Table S3). Former and current smokers reported calling for slightly longer durations than those who had never smoked (Supplementary Table S3; OR for reporting in higher categories for ex-smokers = 1.12, CI, 1.05–1.19; OR for smokers =1.17, CI, 1.08 to 1.27, not shown in the table). After adjustment for age, sex, and education, BMI and handedness were not significantly associated with the duration of calls. With respect to the numbers of calls, most mobile phone users reported making 1 to 4 calls per day (52.9%) (Supplementary Table S4).

The exclusive use of a single mobile phone was the most common usage pattern (Table 2). Overall, 83.9% of participants reported using only one mobile phone. This was more prevalent among individuals over 60 years old (90.2% of men over 60 and 92.3% of women over 60) compared to younger participants (ranging from 71.5% to 84.6% depending on age and sex groups). Additionally, this pattern was more common among women than men. Four out of five participants (80.4%) reported that they did not share their phone with others. Phone sharing was more common among 30–44 year olds compared to other age groups.

Approximately half of the mobile phone users (53.7%) reported using the phone on the right side of their head, and a third (33.3%) reported using it on the left (Table 2). Younger participants reported more right-side use (67.9% of men and 75.1% of women under 30 years old), while the lowest proportion of right-side use was observed in the oldest age group (34.8% of men and 38.4% of women over 60 years old). The use of mobile phones on both sides of the head was similar across all ages, ranging from 7% to 12% across age–sex groups.

**Figure 2:**
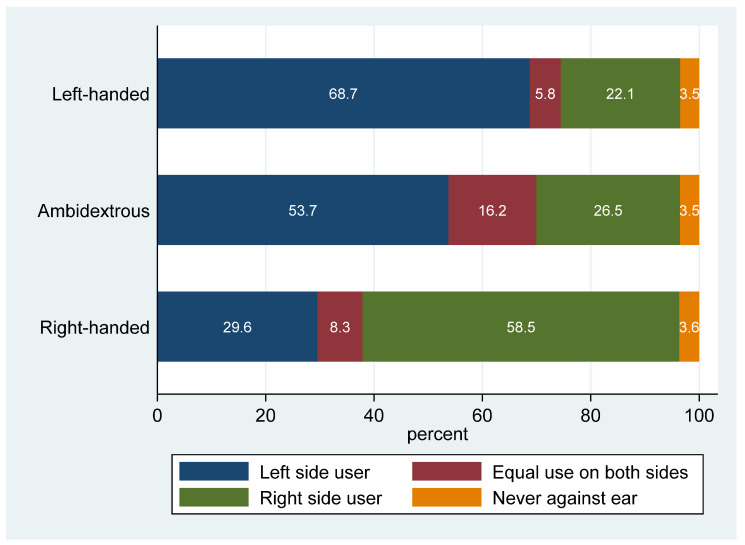
Description of laterality of mobile phone use among left-handed, ambidextrous, and right-handed participants of Cosmos-France, 2017–19. Percentages are shown excluding missing values, presented in white font.

Ambidextrous individuals behaved more similarly to left-handed participants than to right-handed participants regarding the side of mobile phone use (Figure 2). Overall, 10,300 participants (59.1%) used their mobile phone on the same side as their handedness, considering ambidextrous individuals as left-handed (not shown). When examining the associations between age, sex, and side of use by participants’ handedness, we observed that, while the agreement was fairly constant over age for left-handed participants (58.4% to 65.8% left-side users and 20.5% to 27.1% right-side users, depending on age and sex), these proportions varied markedly by age and sex among right-handed participants, with an association between younger age and increased right-side use (Supplementary Figure S6). Among right-handed men, the proportion of right-side users was 77.9% for those under 30 years old and 55.4% for those over 60 years old (p-trend < 0.001). Among right-handed women, the proportions were 81.4% and 50.1%, respectively (p-trend < 0.001).

More than half of the users (53.4%) never or almost never used loudspeakers, hands-free kits, or earphones, particularly older individuals (61.5% among men older than 60 years old, 67.1% among women of the same age group). The use of Voice over Internet Protocol (VoIP) was uncommon (Table 2): overall, 84.9% of users hardly used it (56.3% “did not make calls this way” and 28.3% “used for a few calls”) and 9.2% of users were unsure about their VoIP usage (“does not know the proportion of calls”, do not know, and missing). VoIP usage was nearly twice as high among individuals under 30 years old compared to those over 60.

In analyzing lifetime mobile phone usage, we found that the cessation of mobile phone usage was uncommon. Only 105 participants reported having previously used a mobile phone for voice calls more than once a week but having since discontinued this use. This accounted for 15.2% of non-users (within the three months before the questionnaire) and 0.6% of the total study population. 

Most current mobile phone users (N = 17,245) provided information on when they started using mobile phones (Table 2): 3.7% reported beginning use in the earliest days of mobile telephony in France, i.e., in 1992, 6.0% reported beginning use between 1993 and 1995, and 39.1% reported beginning use between 1996 and 2000. Men generally started using mobile phones earlier than women among participants over 30 years of age, but there was no difference among the youngest participants. Among those aged 30–44 years, 53.5% of men started using mobile phones between 1996 and 2000, compared to 47.7% of women and among older participants,, the difference was similar or larger. Mutually adjusted linear regression models indicated that education, smoking status, and BMI significantly influenced the start of mobile phone use after adjustments for age and sex, but not handedness (Supplementary Table S5). Specifically, people with the lowest education started 1.09 years later than those with the highest education, those who had ever smoked started 1.20 years earlier than those who had never smoked, obese individuals started 0.98 years earlier than those with a healthy weight, and underweight individuals started 0.60 years later than those with a healthy weight.

Participants’ reports on the amount of phone use increased from the start of usage to that reported in 2017–19. In addition, the earlier the participants had started using mobile phones, the more likely they were to report higher levels of use (Supplementary Figure S7).

### 3.2. Use of Cordless Phones

Overall, two-thirds of the participants (66.0%) reported that they used a cordless phone at the time of the interview; this proportion increased with the age of participants and was higher in women (Table 3). The most reported duration of cordless phone use was 5–29 min per week (40.1% overall). The primary determinant of the initiation period for cordless phone use was the participant’s age. For example, individuals aged 45–59 years reported on average first using a cordless phone in the year 2000, while those under 30 years of age on average reported the year 2010. In all age groups, except for those aged 30–44, men reported starting the use of cordless phones slightly earlier than women. For example, among men aged 60 years or older, 31.9% had started using a cordless phone in the 1990s, compared to 25.1% of women in the same age group. Cordless phone use and mobile phone use are related in a complex way (Supplementary Figure S8).

### 3.3. Use of Wireless Connection Networks

Overall, 88.4% of the participants reported using a wireless connection for any equipment (personal computer, laptop, tablet, e-book reader, media player, or video game console), with >92% use of wireless networks among people ≤ 45 years old, and <85% among people ≥ 60 years (Table 4). Among users of wireless networks, most reported using them with a laptop. Men reported slightly higher use than women of the same age for personal computers and laptops. 

## 4. Discussion

In this paper, we present the first descriptive results of self-reported mobile phone use and the use of other RF-emitting equipment in the COSMOS-France study, and their relationships with age, sex, education, smoking, and BMI, as these are all important determinants of future health endpoints, and with handedness. The overall participation in France was the highest among all COSMOS countries and was comparable to response rates observed in studies with a similar design, where a sub-cohort was nested within a larger, ongoing cohort [1,2,3]. The COSMOS-France population was older at recruitment than the COSMOS-Netherlands (54% participants > 50 years old in 2011–12) and COSMOS-UK cohorts (in 2010–12, 20.1% participants > 60 years), but this difference has been almost entirely attenuated by the ageing of the above-mentioned cohorts. The group of individuals under 30 years old at recruitment was small, particularly among men (N = 266), which limits the interpretation of findings for this age and sex group. However, the pooling with the other COSMOS cohorts (N = 37,548 individuals under 30 years old) could help mitigate this limitation when needed [4]. The gender balance is comparable to the COSMOS-UK cohort (55.5% women in France, 52.4% in the UK), whereas in COSMOS-Netherlands, the balance was biassed by design due to partial recruitment from a female nurse cohort [2,3]. With respect to education, COSMOS-France included a proportion of participants with secondary education comparable to COSMOS-UK (22.4% versus 28.4%). The proportion of current and former smokers and those who had never smoked in COSMOS-France was unexpectedly similar to other COSMOS cohorts (for example, current smokers: 13.6% in the French cohort, 13% in the Dutch cohort, and 12.5% in the British cohort). With respect to BMI, the French cohort was like COSMOS-Netherlands (proportion of overweight people: 30.4% (COSMOS-France) and 32% (COSMOS-Netherlands)) but there was less obesity than in the COSMOS-UK cohort (proportion of obese people: 10.6% (COSMOS-France), 12% (COSMOS-Netherlands), and 18.5% (COSMOS-UK)).

Overall, mobile phone reported usage patterns varied sometimes markedly, by sex and age. The age-related patterns, in particular, were obviously influenced by the availability of technologies—namely, 2G (1992), 3G (2001), and 4G (2012)—and their associated costs. Almost all participants reported using a mobile phone in 2017–19; the most common pattern of use was to talk 5–29 min per week on one mobile phone. The typical call duration observed in COSMOS-France was similar to that reported in COSMOS-Denmark (1). Current mobile phone use was higher overall than in COSMOS-Netherlands (72% at baseline had used a mobile phone in their life) and COSMOS-UK (7.1% non-regular users at baseline). The duration of phone use in COSMOS-France was higher than in COSMOS-Netherlands but lower than in COSMOS-UK (users who were on calls for >30 min/week at recruitment: France: 54%, Netherlands 36%, UK: 62.0%). Slightly less than half of COSMOS-France mobile phone users reported having started to use mobile phones by the year 2000, a proportion higher than in COSMOS-Netherlands (approximately 36%) and similar to COSMOS-UK (49.4%). This corresponds to almost 25 years of exposure duration to date and therefore covers the typical induction periods assumed for chronic diseases such as cancer. Education appeared to be related to mobile phone use, with more highly educated participants comprising fewer non-users, and a greater number of intensive users. In Denmark, in 1985, the income of mobile phone users was markedly higher than that of non-users in 1985; by 1995, this disparity had diminished, reflecting the decreasing prices of mobile phone subscriptions [17]. Interestingly, in France during 2017–19, differences in mobile phone usage still persist by education level, potentially related to income, different usage patterns, or both. Smoking was also related to mobile phone use characteristics, with smokers and ex-smokers using their phone more than non-smokers, regardless of their education level. In our population, BMI was related to fewer characteristics of mobile phone use, unlike the association between obesity and mobile phone use reported in COSMOS-UK [2]. Left-handedness has long been known to be associated with addictive behaviours, including smoking, alcohol drinking, and gaming [18,19,20]. However, we did not observe higher self-reported durations of mobile phone use among left-handed or ambidextrous individuals. Although there was some correspondence between handedness and laterality of use, the relationship was not strong. We observed a gradual decrease in right-side use among right-handed individuals with increasing age, which was somewhat more pronounced among females. Our findings were similar to, albeit weaker than, those reported in Japan: right-handed children ≤ 14 years reported 68% right-side use (N = 493), while adults reported 37% right-side use (N = 1287) in a somewhat younger population [21]. These results are in line with other investigations [22,23]. This calls for further caution in the conduct of phone use laterality analyses and in their interpretation, calling for careful consideration of accurate age matching and age adjustments in these analyses.

We observed an increasing trend in the level of use over participants’ lifetime phone history in the COSMOS-France data; a similar observation was made in Finland [24].

Older individuals and women reported more current use of cordless phones, compared to their younger and male counterparts, with the majority of participants under 30 years not using these phones. Among users, women tended to start using cordless phones later than men, possibly reflecting a greater inclination among younger individuals and men toward newer technologies, such as using mobile phones over cordless phones. Although limited comparison data are available, the overall frequency of cordless phone use in France in 2017–19 (66% overall, 42.2 to 68.8% among those aged 30–59 years) was lower than that reported among Germans aged 30–59 in 2002–2005 (76–77% Digital Enhanced Cordless Telephone (DECT) users among controls) [25]. The age trend observed in France was somewhat consistent with pooled DECT-generated RF exposure data for individuals aged 20–50 [13].

We found a significant decrease in self-reported usage of devices connected to Wi-Fi with increasing age, except for e-book readers; we are not aware of any comparison data.

The strengths of this study include its large sample size, enabling subgroup analyses of mobile phone users, and the use of the same questions as the other COSMOS cohorts, facilitating data pooling across studies. The COSMOS-France cohort was established within the framework of the Constances general population cohort study. This setup saved considerable effort and avoided the duplication of work since a wealth of high-quality data had already been collected within the Constances framework. Additionally, this helped reduce the length of the COSMOS questionnaire, which presumably contributed to the good participation rate for a mobile phone study. The COSMOS-France cohort comprises sizable groups of all adult ages, sexes, and education levels and features a wide geographical distribution within France. It provides an excellent view of contemporary mobile phone use in the French population, as well as a comprehensive history of mobile phones and DECT of usage since their inception. The follow-up of our cohort, using repeated questionnaires to capture changes related to the introduction of the 5G network (December 2020 in France), along with detailed information on usage patterns, will enable the evaluation of shifts in participants’ habits over time. COSMOS-France participants were recruited from among Constances participants who had completed at least one follow-up questionnaire to maximize respondent numbers. However, some individuals chose not to participate in the COSMOS-France cohort. While this non-participation may not substantially affect exposure–disease relationship analyses, it could introduce biases in the estimates presented in this publication. The Constances study, which faces a similar challenge, includes methods to adjust certain estimates to better reflect the general population; however, applying these adjustments is beyond the scope of the current work [26]. In this analysis, education, BMI, and smoking status were collected an average of 4.4 years before mobile phone details, so this slightly outdated information may have introduced some inaccuracies. The main limitation of COSMOS-France, as with other COSMOS components and studies that rely on self-reported mobile phone use, is the difficulty in accurately recalling mobile phone use. This issue has been observed in both case–control [5] and cohort studies [27]. Errors in the reported amount of use are unavoidable, and extensive simulations of the consequences of these errors have been conducted using case–control datasets [8], showing that biases may create spurious associations between mobile phone use and brain tumour risk. One main source of error, however, is avoided in the cohort design: since mobile phone use is assessed before the onset of the disease of interest, the reporting of exposure is not influenced by the disease, unlike in case–control studies. COSMOS, in general, obtains information from network operators to complement the self-reported mobile phone use data. Both methods have advantages and disadvantages, but together they provide better insights into the nature of possible bias [4].

## 5. Conclusions

COSMOS is the only cohort specifically designed to investigate the relationship between mobile phone use and health. In this initial description of the French component, COSMOS-France, we outlined the sociodemographic characteristics, smoking status, BMI, and handedness of the cohort and provided a contemporary overview of self-reported mobile phone usage, including frequency, methods of use, and the use of cordless phones and other Wi-Fi-enabled devices in France. These insights will be valuable for future analyses, allowing comparisons with other European COSMOS cohorts and with other studies.

## Figures and Tables

**Table 1:** Sociodemographic characteristics, smoking status, and BMI in the Cosmos-France study population, France, 2017–2019.

	Participants in the Cosmos-France Study *	
Characteristics	N	%
analyzed dataset *	18,502	100
Sex and age (years)		
Men < 30	266	1.4
Women < 30	507	2.7
Men 30–44	1611	8.7
Women 30–44	2327	12.6
Men 45–59	2866	15.5
Women 45–59	3510	19.0
Men 60 and above	3497	18.9
Women 60 and above	3918	21.2
Education duration (years)		
≤11	4135	22.4
12–13	3052	16.5
14–15	4826	26.1
16	1732	9.4
17 or more	4486	24.3
Other, missing	271	1.5
Smoking status		
Never smoked	8828	47.7
Former smoker	6404	34.6
Current smoker	2523	13.6
Other, missing	747	4.0
Body mass index		
Underweight	452	2.4
Healthy weight	10,183	55.0
Overweight	5616	30.4
Obese	1967	10.6
Missing	284	1.5

Note: * participants who returned the baseline questionnaire.

**Table 2:** Description of call time and patterns in phone usage in 2017–2019, Cosmos-France study.

	Age Groups Among Men								Age Groups Among Women									
	<30 Years		30–44 Years		45–59 Years		60+ Years		<30 Years		30–44 Years		45–59 Years		60+ Years		Total	
	N	%	N	%	N	%	N	%	N	%	N	%	N	%	N	%	N	%
Total	266	100	1611	100	2866	100	3497	100	507	100	2327	100	3510	100	3914	100	18,502	100
Current mobile phone users																		
Yes	259	97.4	1577	97.9	2765	96.5	3279	93.8	498	98.2	2275	97.8	3406	97.0	3723	95.0	17,782	96.1
No	7	2.6	32	2.0	94	3.3	209	6.0	9	1.8	51	2.2	100	2.9	189	4.8	691	3.7
Missing	0	0.0	2	0.1	7	0.2	9	0.3	0	0.0	1	0.0	4	0.1	6	0.2	29	0.2
Duration of voice calls (among current mobile phone users)																		
<5 min/week	18	7.0	82	5.2	244	8.8	516	15.7	18	3.6	106	4.7	249	7.3	311	8.4	1544	8.7
5–29 min/week	87	33.6	487	30.9	1022	37.0	1516	46.2	126	25.3	679	29.9	1198	35.2	1485	39.9	6600	37.1
30–59 min/week	65	25.1	321	20.4	573	20.7	653	19.9	136	27.3	544	23.9	745	21.9	886	23.8	3923	22.1
1–3 h/week	69	26.6	413	26.2	573	20.7	461	14.1	153	30.7	646	28.4	819	24.1	795	21.4	3929	22.1
4–6 h/week	14	5.4	162	10.3	215	7.8	94	2.9	45	9.0	200	8.8	265	7.8	179	4.8	1174	6.6
≥7 h/week	6	2.3	111	7.0	138	5.0	33	1.0	20	4.0	100	4.4	129	3.8	61	1.6	598	3.4
Missing	0	0.0	1	0.1	0	0.0	6	0.2	0	0.0	0	0.0	1	0.0	6	0.2	14	0.1
Number of phones (among current mobile phone users)																		
1 phone	205	79.2	1127	71.5	2094	75.7	2959	90.2	403	80.9	1816	79.8	2881	84.6	3436	92.3	14,921	83.9
2 or more phones	54	20.9	445	28.2	651	23.5	303	9.2	92	18.5	447	19.7	504	14.8	260	7.0	2756	15.5
Missing, uninterpretable	0	0.0	5	0.3	20	0.7	17	0.5	3	0.6	12	0.5	22	0.7	26	0.7	105	0.6
Sharing of phones (among current mobile phone users)																		
Never or almost never for all phones	215	83.0	1256	79.6	2312	83.6	2606	79.5	382	76.7	1662	73.1	2786	81.8	3076	82.6	14,295	80.4
At least sometimes for at least 1 of the phones	40	15.4	303	19.2	413	14.9	613	18.7	110	22.1	593	26.1	577	16.9	580	15.6	3229	18.2
Missing	4	1.5	18	1.1	40	1.5	60	1.8	6	1.2	20	0.9	43	1.3	67	1.8	258	1.5
Laterality while calling (among current mobile phone users)																		
Most often against left ear	45	17.4	398	25.2	939	34.0	1140	34.8	69	13.9	631	27.7	1276	37.5	1428	38.4	5926	33.3
Equally on both sides	30	11.6	143	9.1	239	8.6	290	8.8	47	9.4	189	8.3	252	7.4	301	8.1	1491	8.4
Most often against right ear	176	68.0	986	62.5	1473	53.3	1666	50.8	373	74.9	1373	60.4	1733	50.9	1779	47.8	9559	53.8
Never close to ear	7	2.7	40	2.5	80	2.9	159	4.9	6	1.2	60	2.6	105	3.1	179	4.8	636	3.6
Missing	1	0.4	10	0.6	34	1.2	24	0.7	3	0.6	22	1.0	40	1.2	36	1.0	170	1.0
Use of headset or speaker (among current mobile phone users)																		
Never	92	35.5	634	40.2	1398	50.6	2016	61.5	156	31.3	926	40.7	1781	52.3	2499	67.1	9502	53.4
Less than or half of the calls	124	47.9	699	44.3	992	35.9	913	27.8	249	50.0	1003	44.1	1147	33.7	793	21.3	5920	33.3
More than half or all the calls	43	16.6	229	14.5	348	12.6	314	9.6	90	18.1	319	14.0	432	12.7	365	9.8	2140	12.0
Missing	0	0.0	15	1.0	27	1.0	36	1.1	3	0.6	27	1.2	46	1.4	66	1.8	220	1.2
Use of the Voice over Internet Protocol—VoIP (among current mobile phone users)																		
None or a few calls	213	82.2	1362	86.4	2382	86.2	2789	85.1	402	80.7	1939	85.2	2865	84.1	3136	84.2	15,088	84.9
Half of the calls	21	8.1	78	5.0	107	3.9	120	3.7	40	8.0	122	5.4	164	4.8	161	4.3	813	4.6
All the calls	4	1.5	21	1.3	30	1.1	43	1.3	13	2.6	34	1.5	51	1.5	58	1.6	254	1.4
Does not know, not sure, missing	21	8.1	116	7.4	246	8.9	327	10.0	43	8.6	180	7.9	326	9.6	368	9.9	1627	9.2
Year of start of use (among current mobile phone users who provided information about the year that they started using a mobile phone)																		
1992	0	0.0	6	0.4	102	3.8	298	9.5	1	0.2	9	0.4	82	2.5	132	3.7	630	3.7
1993–95	0	0.0	22	1.4	253	9.4	383	12.2	0	0.0	32	1.4	167	5.0	184	5.2	1041	6.0
1996–00	6	2.4	835	53.5	1272	47.0	1139	36.3	6	1.2	1069	47.7	1316	39.5	1102	31.2	6745	39.1
2001–05	63	24.8	548	35.1	523	19.3	548	17.5	128	26.0	825	36.8	796	23.9	822	23.3	4253	24.7
2006–10	134	52.8	102	6.5	362	13.4	451	14.4	262	53.1	217	9.7	577	17.3	762	21.6	2867	16.6
2011–15	46	18.1	34	2.2	139	5.1	209	6.7	76	15.4	61	2.7	265	8.0	343	9.7	1173	6.8
2016–19	5	2.0	14	0.9	53	2.0	108	3.4	20	4.1	28	1.3	125	3.8	183	5.2	536	3.1
Missing	5	NA	16	NA	61	NA	143	NA	5	NA	34	NA	78	NA	195	NA	537	NA

**Table 3:** Duration of call time and period of the start of use of cordless phones by age and sex in 2017–2019, Cosmos-France study.

	Age Groups Among Men								Age Groups Among Women									
	<30 Years		30–44 Years		45–59 Years		60+ Years		<30 Years		30–44 Years		45–59 Years		60+ Years		Total	
	N	%	N	%	N	%	N	%	N	%	N	%	N	%	N	%	N	%
Total	266	100	1611	100	2866	100	3497	100	507	100	2327	100	3510	100	3914	100	18,502	100
Current cordless phone use																		
Yes	104	39.1	680	42.2	1857	64.8	2644	75.6	206	40.6	1218	52.3	2413	68.8	3094	79.0	12,216	66.0
No	162	60.9	921	57.2	984	34.3	804	23.0	295	58.2	1089	46.8	1061	30.2	766	19.6	6082	32.9
Missing	0	0.0	10	0.6	25	0.9	49	1.4	6	1.2	20	0.9	36	1.0	58	1.5	204	1.1
Duration of voice calls (cordless phone users only)																		
<5 min/week	15	14.4	113	16.6	337	18.2	446	16.9	40	19.4	164	13.5	284	11.8	290	9.4	1689	13.8
5–29 min/week	35	33.7	260	38.2	756	40.7	1288	48.7	60	29.1	396	32.5	887	36.8	1220	39.4	4902	40.1
30–59 min/week	20	19.2	132	19.4	371	20.0	520	19.7	34	16.5	290	23.8	574	23.8	772	25.0	2713	22.2
1–3 h/week	21	20.2	106	15.6	239	12.9	279	10.6	38	18.5	226	18.6	464	19.2	584	18.9	1957	16.0
≥4 h/week	10	9.6	58	8.5	108	5.8	63	2.4	29	14.1	126	10.3	163	6.8	135	4.4	692	5.7
Missing duration	3	2.9	11	1.6	46	2.5	48	1.8	5	2.4	16	1.3	41	1.7	93	3.0	263	2.2
Start of use (cordless phone users only)																		
≤1989	0	0	0	0	57	3.1	166	6.3	0	0	3	0.3	55	2.3	169	5.5	450	3.7
1990–99	1	0.5	124	18.2	615	33.1	843	31.9	1	0.5	216	17.7	735	30.5	776	25.1	3314	27.1
2000–09	71	34.5	349	51.3	807	43.5	1062	40.2	70	34.2	673	55.3	998	41.4	1185	38.3	5185	42.4
≥2010	106	51.5	136	20.0	205	11.0	313	11.8	106	51.7	195	16.0	308	12.8	445	14.4	1756	14.4
Missing start year	28	13.6	71	10.4	173	9.3	260	9.8	28	13.7	131	10.8	317	13.1	519	16.8	1511	12.4

Note: cordless phones were described to the participants as a phone of limited range allowing the user to move around at home or at work. The following abbreviations were used: min for minute, h for hour.

**Table 4:** Use of wireless networks overall and for each type of equipment by age and sex in 2017–2019, COSMOS-France study.

	Age Groups Among Men								Age Groups Among Women									
	<30 Years		30–44 Years		45–59 Years		60+ Years		<30 Years		30–44 Years		45–59 Years		60+ Years		Total	
	N	%	N	%	N	%	N	%	N	%	N	%	N	%	N	%	N	%
Total	266	100	1611	100	2866	100	3497	100	507	100	2327	100	3510	100	3914	100	18,502	100
Wireless network use over the last 3 months																		
Yes	249	93.6	1491	92.6	2541	88.7	2914	83.3	470	92.7	2149	92.4	3209	91.4	3324	84.8	16,347	88.4
No	15	5.6	114	7.1	297	10.4	534	15.3	34	6.7	157	6.8	276	7.9	516	13.2	1943	10.5
Missing	2	0.8	6	0.4	28	1.0	49	1.4	3	0.6	21	0.9	25	0.7	78	2.0	212	1.2
Duration of wireless network use with personal computers (wireless network users only)																		
None or almost	178	71.5	1007	67.5	1506	59.3	1516	52.0	321	68.3	1375	64.0	1748	54.5	1853	55.8	9504	58.1
<1 h/day	22	8.8	190	12.7	473	18.6	590	20.3	49	10.4	321	14.9	626	19.5	766	23.0	3037	18.6
1–3 h/day	18	7.2	173	11.6	387	15.2	677	23.2	39	8.3	210	9.8	468	14.6	585	17.6	2557	15.6
≥4 h/day	31	12.5	120	8.1	175	6.9	130	4.5	61	13.0	242	11.3	367	11.4	120	3.6	1246	7.6
Missing	0	0.0	1	0.1	0	0.0	1	0.0	0	0.0	1	0.1	0	0.0	0	0.0	3	0.0
Duration of wireless network use with laptops (wireless network users only)																		
None or almost	38	15.26	278	18.65	711	28.0	1212	41.6	82	17.45	515	24.0	1121	34.9	1515	45.6	5472	33.5
<1 h/day	51	20.5	422	28.3	682	26.8	705	24.2	150	31.9	740	34.4	984	30.7	885	26.6	4619	28.3
1–3 h/day	83	33.3	418	28.0	718	28.3	827	28.4	143	30.4	551	25.6	759	23.7	779	23.4	4278	26.2
≥4 h/day	77	30.9	371	24.9	430	16.9	169	5.8	95	20.2	343	16.0	343	10.7	144	4.3	1972	12.1
Missing	0	0.0	2	0.1	0	0.0	1	0.0	0	0.0	0	0.0	2	0.1	1	0.0	6	0.0
Duration of wireless network use with tablets (wireless network users only)																		
None or almost	179	71.89	826	55.4	1450	57.1	1863	63.9	348	74.04	1237	57.6	1811	56.4	2019	60.7	9733	59.5
<1 h/day	36	14.5	392	26.3	640	25.2	615	21.1	68	14.5	569	26.5	770	24.0	604	18.2	3694	22.6
1–3 h/day	24	9.6	235	15.8	410	16.1	392	13.5	46	9.8	298	13.9	567	17.7	641	19.3	2613	16.0
≥4 h/day	10	4.0	38	2.6	41	1.6	44	1.5	8	1.7	45	2.1	61	1.9	60	1.8	307	1.9
Duration of wireless network use with e-book readers (wireless network users only)																		
None or almost	240	96.39	1422	95.37	2450	96.4	2791	95.8	450	95.74	2006	93.4	2991	93.2	3079	92.6	15,429	94.4
<1 h/day	8	3.2	59	4.0	74	2.9	83	2.9	15	3.2	107	5.0	167	5.2	160	4.8	673	4.1
1–3 h/day	1	0.4	7	0.5	17	0.7	34	1.2	5	1.1	36	1.7	51	1.6	78	2.4	229	1.4
≥4 h/day	0	0.0	3	0.2	0	0.0	6	0.2	0	0.0	0	0.0	0	0.0	7	0.2	16	0.1
Duration of wireless network use with portable media players (wireless network users only)																		
None or almost	229	91.97	1423	95.44	2441	96.1	2833	97.2	446	94.89	2056	95.7	3072	95.7	3250	97.8	15,750	96.4
<1 h/day	15	6.0	49	3.3	74	2.9	64	2.2	16	3.4	63	2.9	104	3.2	61	1.8	446	2.7
1–3 h/day	4	1.6	15	1.0	24	0.9	15	0.5	7	1.5	26	1.2	28	0.9	12	0.4	131	0.8
≥4 h/day	1	0.4	4	0.3	2	0.1	2	0.1	1	0.2	4	0.2	5	0.2	1	0.0	20	0.1
Duration of wireless network use with portable video game consoles (wireless network users only)																		
None or almost	198	79.52	1370	91.88	2472	97.3	2895	99.4	427	90.85	2057	95.7	3169	98.8	3310	99.6	15,898	97.3
<1 h/day	27	10.8	86	5.8	47	1.9	11	0.4	30	6.4	73	3.4	31	1.0	8	0.2	313	1.9
1–3 h/day	21	8.4	29	2.0	19	0.8	7	0.2	12	2.6	17	0.8	8	0.3	5	0.2	118	0.7
≥4 h/day	3	1.2	6	0.4	3	0.1	1	0.0	1	0.2	2	0.1	1	0.0	1	0.0	18	0.1

Note: duration reported for the use of equipment during the week. The abbreviation h is used for hour.

## Data Availability

The datasets presented in this article are not readily available because of privacy, legal and ethical reasons. Requests to access the datasets should be directed to corresponding author. Where authors are identified as personnel of the International Agency for Research on Cancer/World Health Organization, the authors alone are responsible for the views expressed in this article, and they do not necessarily represent the decisions, policy, or views of the International Agency for Research on Cancer/World Health Organization.

## Supplementary Materials

The following supporting information can be downloaded at: https://www.mdpi.com/article/10.3390/ijerph21111514/s1, Material S1: Criteria for inclusion in the pool of Constances volunteers from which COSMOS-France participants were selected; Material S2: COSMOS-France questionnaire; Figure S1: Distribution of Education Duration: Stacked Percentages of Individuals by Years of Education within Each Age and Sex Category, COSMOS-France Cohort, 2017–19 (N = 18,231); Figure S2: Distribution of Smoking Status: Stacked Percentages of Never Smokers, Ex-Smokers, and Smokers within Each Age and Sex Category, COSMOS-France Cohort, 2017–2019 (N = 17,755); Figure S3: Distribution of Body Mass Index (BMI): Stacked Percentages of Underweight, Healthy Weight, Overweight, and Obese Individuals within Each Age and Sex Category, COSMOS-France Cohort, 2017–19 (N = 18,218); Figure S4: Distribution of Handedness: Stacked Percentages of Left-Handed, Ambidextrous and Right-Handed Individuals within Each Age and Sex Category, COSMOS-France Cohort, 2017–19 (N = 18,296); Figure S5: Proportion of self-reported duration of mobile phone use by age group for a) men and b) women in the COSMOS-France Cohort, 2017–19; Figure S6: Distribution of Laterality of Mobile Phone Use by Handedness: Stacked Percentages of Left-Side, Equal-Side, Right-Side Mobile Phone Users, and Users Who Never Hold Their Phone Close to Their Head within Each Age and Sex Category among Left-Handed and Ambidextrous and among Right -Handed Participants COSMOS-France cohort, 2017–2019 (N = 18,296); Figure S7: Distribution of Weekly Mobile Phone Usage (stacked proportion of each category) over Participants’ Lifetime Usage History, by Period of First Use, for (a) men and (b) women, COSMOS-France cohort, 2017–19; Figure S8: Distribution Cordless Phone (DECT) Usage and Duration: Stacked Percentages of No Use and Duration of Cordless Phone Usage within Categories of Mobile Phone Use Duration, COSMOS-France Cohort, 2017–19 (N = 17,351); Table S1: Overall participation by age and sex in the COSMOS-France study, 2017–19; Table S2: Odds ratio (OR) and 95% confidence interval (CI) of no use of mobile phone associated with education, smoking, body mass index, and handedness, COSMOS France, 2017–19; Table S3: Predicted probabilities of duration of use, for mobile phone users, according to education length, smoking status, body mass index and handedness, COSMOS France, 2017–19; Table S4: Description of frequency of calls among mobile phone users in 2017–19, COSMOS-France study; Table S5: Effect of education, smoking status, body mass index and handedness on year of start of mobile phone use among mobile phone users, COSMOS-France study, 2017–2019.
