# Supplementary material for: Use of Mobile Phones and Radiofrequency-Emitting Devices in the COSMOS-France Cohort
Source: Int J Environ Res Public Health. 2024 Nov 14;21(11):1514. doi: 10.3390/ijerph21111514 (PMC11593438; doi:10.3390/ijerph21111514)
Supplement: Supplementary file 1 [file ijerph-21-01514-s001.zip › ijerph-3251228-supplementary.pdf]

Supplementary material to : Use of Mobile Phones and Radiofrequency-Emitting Devices in the COSMOS-France Cohort

Authors

Isabelle Deltour [1*], Florence Guida [1], Céline Ribet [2], Marie Zins [2], Marcel Goldberg [2], Joachim Schüz [1]

Authors Affiliations:

1. Environment and Lifestyle Epidemiology Branch, International Agency for Research on Cancer (IARC/WHO), 69366 Lyon, France; guidaf@iarc.who.int (F.G.); schuzj@iarc.who.int (J.S.)
2. Université Paris Cité, Paris Saclay University, UVSQ, INSERM UMS 011, 94800 Villejuif, France; celine.ribet@inserm.fr (C.R.); marie.zins@inserm.fr (M.Z.); marcel.goldberg@inserm.fr (M.G.)

*Correspondence: deltouri@iarc.who.int
Dr Isabelle Deltour
Environment and Lifestyle Epidemiology Branch
International Agency for Research on Cancer / World Health Organization (IARC/WHO)
25 avenue Tony Garnier CS 90627
69366 Lyon Cedex 07, France

Supplementary Material S1: Criteria for inclusion in the pool of Constances volunteers from which COSMOS-France participants were selected.

For the pilot of COSMOS-France, the pool of Constances volunteers from which COSMOS-France participants were randomly selected consisted of individuals who:

- Had been recruited in Constances between 2012 and 2015,
- Had answered at least once to an annual Constances follow-up questionnaire (follow-up from 2013 to 2016),
- Were not participants in Constances pilot recruitment phase,
- Were not included in another study whose data collection was during the same period than COSMOS data collection.

For the main recruitment phase, the pool of Constances volunteers from which COSMOS-France participants were randomly selected consisted of individuals who:

- Had been recruited in Constances between 2012 and 2016,
- Had answered at least once to an annual Constances follow-up questionnaire (follow-up from 2013 to 2017),
- Were not participants in Constances pilot recruitment phase,
- Had not been invited to participate to COSMOS_France pilot study,
- Were not included in another study whose data collection was during the same period than COSMOS-France data collection.

For the main recruitment phase, the subsample of participants who were sent a paper questionnaire included 30,000 volunteers randomly selected out of the 43,067 Constances volunteers included in the 2018 paper version of the follow-up of Constances. The subsample of participants who were first invited to answer to the COSMOS-France questionnaire via the internet, included 10,000 volunteers randomly selected out of the 29,939 Constances volunteers included in the 2018 internet follow-up of Constances.

Supplementary Material S2: COSMOS-France questionnaire.

- ***Êtes-vous droitier, gaucher ou ambidextre ?*** ☐ Droitier ☐ Gaucher ☐ Ambidextre

I. VOTRE UTILISATION DE TÉLÉPHONES MOBILES

Répondez aux questions suivantes en tenant compte de **tous vos appels avec un téléphone mobile** (portable ou smartphone) même s'ils ne sont pas sur votre facture téléphonique, comme par exemple les appels que vous recevez, les appels inclus gratuitement dans votre forfait, les appels avec un service VoIP comme Skype, Viber, WhatsApp…, les appels passés ou reçus avec le mobile d'une autre personne, les appels professionnels.

A. Votre utilisation habituelle de téléphones mobiles au cours des 3 derniers mois

1. *Au cours des **3 derniers mois**, avez-vous été en communication lors d'appels passés ou reçus avec un mobile au moins une fois par semaine ?*

☐ Oui ☐ Non

➥ Si **non**, passez directement à la question 15, page 7.

2. *Au cours des **3 derniers mois**, combien d'appels par jour en moyenne avez-vous passés ou reçus avec un mobile ?*

Moins d'un appel par jour	1 à 4 appels par jour	5 à 9 appels par jour	10 à 29 appels par jour	30 appels ou plus par jour
☐	☐	☐	☐	☐
			Soit l'équivalent de 1 à 3 appels par heure, pendant 10 heures par jour	*Soit l'équivalent de 3 appels ou plus par heure pendant 10 heures par jour*

3. *Au cours des **3 derniers mois**, combien de temps par semaine en moyenne avez-vous passé en communication avec un mobile lors d'appels émis ou reçus ?*

Moins de 5 minutes par semaine	5 à 29 minutes par semaine	30 à 59 minutes par semaine	1 à moins de 4 heures par semaine	4 à moins de 7 heures par semaine	7 heures ou plus par semaine
☐	☐	☐	☐	☐	☐
			Soit l'équivalent de 8 à 34 minutes par jour	*Soit l'équivalent de 35 à 59 minutes par jour*	*Soit l'équivalent de 1 heure ou plus par jour*

4. Habituellement, contre quelle oreille tenez-vous le mobile lorsque vous êtes en communication ?

- ☐ Je ne tiens jamais de téléphone mobile contre mon oreille
- ☐ Le plus souvent à droite
- ☐ Le plus souvent à gauche
- ☐ Autant à droite qu'à gauche

*5. Au cours des **3 derniers mois**, combien de temps en moyenne avez-vous passé en communication avec un mobile sans le tenir contre votre oreille lors d'appels émis ou reçus (en utilisant un kit main-libre ou Bluetooth (oreillette ou casque), le haut-parleur, des écouteurs ou en passant des appels vidéo) ?*

Jamais ou presque jamais	Moins de la moitié du temps total de conversation	Environ la moitié du temps total de conversation	Plus de la moitié du temps total de conversation	Presque toujours ou toujours
☐	☐	☐	☐	☐

➥ Si **jamais ou presque jamais**, passez directement à la question 8, page 4.

*6. Au cours des **3 derniers mois,** lorsque vous étiez en communication avec un mobile sans le tenir contre votre oreille, où était-il placé le plus souvent ?*

- ☐ Au niveau du buste (par exemple poche de poitrine ou suspendu autour du cou)
- ☐ Région antérieure des hanches (par exemple poche avant du pantalon ou de la jupe, à la ceinture à l'avant ou sur les genoux)
- ☐ Région postérieure des hanches (par exemple poche arrière du pantalon ou de la jupe ou à la ceinture, dans le dos)
- ☐ Dans ma main
- ☐ Ailleurs près de mon corps
- ☐ Ailleurs mais pas en contact avec mon corps, par exemple posé sur la table

*7. Au cours des **3 derniers mois**, et sur la totalité des appels passés et reçus avec un mobile, combien de temps en moyenne avez-vous utilisé une **oreillette sans fil ou un casque sans fil** (technologie Bluetooth ou main-libre) ?*

Jamais ou presque jamais	Moins de la moitié du temps total de conversation	Environ la moitié du temps total de conversation	Plus de la moitié du temps total de conversation	Presque toujours ou toujours
☐	☐	☐	☐	☐

*8. Au cours des **3 derniers mois,** avez-vous utilisé un service VoIP comme Skype, Viber, WhatsApp… pour passer ou recevoir des appels sur un mobile ? (La téléphonie VoIP – voix sur IP - fait transiter l'appel vocal par Internet).*

- ☐ Oui, presque tous mes appels sont passés de cette façon
- ☐ Oui, presque la moitié de mes appels sont passés de cette façon
- ☐ Oui, mais seulement quelques appels sont passés de cette façon
- ☐ Oui, mais je ne connais pas la proportion d'appels que je passe de cette façon
- ☐ Non, je ne passe aucun appel de cette façon
- ☐ Je ne sais pas

➥ Si **non** ou **je ne sais pas**, passez directement à la question 10, page 4.

9. Laquelle de ces propositions décrit le mieux la façon dont vous utilisez un service VoIP avec un mobile pour passer ou recevoir des appels ?

- ☐ Uniquement quand un réseau WI-FI est disponible
- ☐ Uniquement quand un réseau mobile (2G, 3G, 4G, Edge,…) est disponible
- ☐ L'une ou l'autre, cela dépend de la connexion disponible à ce moment-là
- ☐ Je ne sais pas

*10. Au cours des **3 derniers mois**, combien de SMS par jour en moyenne avez-vous envoyé(s) avec un mobile ? (en excluant les messages instantanés et le tchat)*

Jamais ou je n'envoie pas de SMS	Moins d'1 SMS par jour	1 à 4 SMS par jour	5 à 9 SMS par jour	10 à 19 SMS par jour	20 à 29 SMS par jour	30 SMS ou plus par jour
☐	☐	☐	☐	☐	☐	☐

*11. Au cours des **3 derniers mois**, combien de fois par jour en moyenne avez-vous consulté vos e-mails, téléchargé de la musique ou des vidéos, ou accédé à Internet avec un mobile ?*

- ☐ Le mobile télécharge en permanence (push e-mails par exemple)
- ☐ De nombreuses fois par jour
- ☐ Une ou deux fois par jour
- ☐ Rarement
- ☐ Jamais ou ne s'applique pas

*12. Au cours des **3 derniers mois**, combien de temps par jour en moyenne avez-vous utilisé un mobile autrement que pour téléphoner (par exemple pour envoyer des SMS, tchatter, consulter vos e-mails, accéder à Internet, participer aux réseaux sociaux, télécharger de la musique/des vidéos, …) ?*

Moins de 5 minutes par jour	5 à 29 minutes par jour	30 à 59 minutes par jour	1 à 3 heures par jour	Plus de 3 heures par jour
☐	☐	☐	☐	☐

*13. Au cours des **3 derniers mois**, en tenant compte de votre usage personnel et professionnel, combien de téléphones mobiles avez-vous utilisés au moins une fois par semaine ?*

mobile(s)

14. *Indiquez les 6 premiers chiffres du numéro des 2 téléphones mobiles que vous avez utilisés le plus au cours des **3 derniers mois** pour passer ou recevoir des appels, y compris pour votre travail (Si vous n'avez utilisé qu'un mobile, laissez la colonne de droite vide.) :*

	Mobile 1	Mobile 2
	0 _ _ _ _ _	0 _ _ _ _ _
Je ne souhaite pas communiquer les 6 premiers chiffres de mon numéro de mobile	☐	☐

- *Quel est l'opérateur actuel ?*

Orange ou Sosh	☐	☐
Bouygues Telecom ou B&YOU	☐	☐
SFR ou RED by SFR	☐	☐
Free Mobile	☐	☐
Autre opérateur	☐	☐
Je ne sais pas, je ne souhaite pas répondre	☐	☐

- *Au cours des **3 derniers mois**, à quelle fréquence d'autres personnes ont-elles utilisé ce mobile ?*

Jamais ou presque jamais	☐	☐
Parfois	☐	☐
Souvent	☐	☐

- *Le forfait de ce mobile est :*

Un forfait à votre nom	☐	☐
Un forfait au nom d'une autre personne	☐	☐
Des cartes prépayées	☐	☐
Un forfait professionnel	☐	☐

- *Au cours des **3 derniers mois**, en additionnant tout le temps passé en conversation sur un téléphone mobile, vous avez parlé :*

	Sur le mobile 1	Sur le mobile 2
De 90 à 100 %	☐	☐
De 60 à 89 %	☐	☐
De 30 à 59 %	☐	☐
De 10 à 29 %	☐	☐
Moins de 10 %	☐	☐

🛈 Les questions suivantes concernent l’utilisation des 2 téléphones mobiles que vous venez d’indiquer. Répondez aux questions pour le mobile 1 dans la colonne de gauche et pour le mobile 2 dans la colonne de droite. Si vous n’avez utilisé qu’un seul mobile, laissez la colonne de droite vide.

	**Mobile 1**	**Mobile 2**

- *Ce mobile peut-il se connecter au réseau 3G ? (Lorsque le réseau 3G est connecté, le symbole 3G est visible sur l’écran.)*

	Mobile 1	Mobile 2
Oui	☐	☐
Non	☐	☐
Je ne sais pas	☐	☐

- *Ce mobile peut-il se connecter au réseau 4G ? (Lorsque le réseau 4G est connecté, le symbole 4G est visible sur l’écran.)*

	Mobile 1	Mobile 2
Oui	☐	☐
Non	☐	☐
Je ne sais pas	☐	☐

B. Votre historique d’utilisation de téléphones mobiles

🛈 Les questions suivantes portent sur l’historique de votre utilisation de téléphones mobiles (appels passés et reçus). Si vous n’êtes pas certain(e) de la réponse exacte, indiquez celle qui vous semble s’en approcher le plus.

15. *En quelle année avez-vous commencé à passer ou recevoir des appels avec un téléphone mobile **au moins une fois par semaine** ?*

En : A A A A ☐ Je n’ai jamais utilisé un téléphone mobile aussi souvent

➥ Si **vous n’avez jamais utilisé un mobile aussi souvent**, passez directement à la question 21, page 8.

*16. Au cours des **années indiquées ci-dessous**, combien d'appels en moyenne avez-vous passés ou reçus avec un mobile ?*

	Jamais ou moins d'un appel par semaine	1 à 6 appels par semaine	1 à 4 appels par jour	5 à 9 appels par jour	10 à 29 appels par jour	30 appels ou plus par jour
En 2015	☐	☐	☐	☐	☐	☐
En 2010	☐	☐	☐	☐	☐	☐
En 2005	☐	☐	☐	☐	☐	☐
En 2000	☐	☐	☐	☐	☐	☐
En 1995	☐	☐	☐	☐	☐	☐
En 1990	☐	☐	☐	☐	☐	☐

*17. Au cours des **années indiquées ci-dessous**, combien de temps par semaine en moyenne avez-vous passé en communication lors d'appels émis ou reçus avec un mobile ?*

	Jamais	Moins de 5 minutes par semaine	5 à 29 minutes par semaine	30 à 59 minutes par semaine	1 à 3 heures par semaine	4 à 6 heures par semaine	7 heures ou plus par semaine
En 2015	☐	☐	☐	☐	☐	☐	☐
En 2010	☐	☐	☐	☐	☐	☐	☐
En 2005	☐	☐	☐	☐	☐	☐	☐
En 2000	☐	☐	☐	☐	☐	☐	☐
En 1995	☐	☐	☐	☐	☐	☐	☐
En 1990	☐	☐	☐	☐	☐	☐	☐

18. En quelle année avez-vous commencé pour la première fois à utiliser les réseaux 3G ou 4G ?

- Réseau 3G : en 20 A A ☐ Je n'utilise pas le réseau 3G
- Réseau 4G : en 20 A A ☐ Je n'utilise pas le réseau 4G

19. *Au cours des* ***années indiquées ci-dessous****, combien de temps en moyenne avez-vous utilisé un mobile pour passer ou recevoir des appels sans le tenir contre votre oreille (en utilisant un kit main-libre ou Bluetooth (oreillette ou casque), le haut-parleur, des écouteurs ou en passant des appels vidéo) ?*

	Jamais, presque jamais ou pas d'utilisation de mobile cette année-là	Moins de la moitié du temps total de conversation	Environ la moitié du temps total de conversation	Plus de la moitié du temps total de conversation	Presque toujours ou toujours
En 2015	☐	☐	☐	☐	☐
En 2010	☐	☐	☐	☐	☐
En 2005	☐	☐	☐	☐	☐
En 2000	☐	☐	☐	☐	☐
En 1995	☐	☐	☐	☐	☐
En 1990	☐	☐	☐	☐	☐

20. ***Depuis que vous utilisez un téléphone mobile****, avez-vous changé l'oreille contre laquelle vous le tenez habituellement ?*

- ☐ Non, je n'ai pas fait de changement
- ☐ Oui, dans le passé je ne tenais pas le mobile contre mon oreille
- ☐ Oui, dans le passé j'utilisais plutôt le côté droit
- ☐ Oui, dans le passé j'utilisais plutôt le côté gauche
- ☐ Oui, dans le passé j'utilisais autant le mobile à droite qu'à gauche

➥ Si **non**, passez directement à la question 21, page 9.

➥ Si **oui** :

- En quelle année ce changement a-t-il eu lieu ? Année : A A A A
- À quoi ce changement est-il dû ?
  - ☐ À des problèmes d'audition
  - ☐ À des maux de tête
  - ☐ À une autre raison médicale
  - ☐ À une raison esthétique (par exemple piercing dans l'oreille)
  - ☐ À une autre raison ou sans raison particulière

II. VOTRE UTILISATION DE TÉLÉPHONES SANS FIL

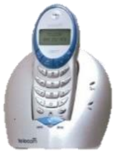

Un téléphone sans fil est un téléphone ayant une portée limitée, qui permet de se déplacer dans une habitation ou une entreprise tout en l'utilisant. Les questions posées concernent l'utilisation que vous avez du téléphone sans fil, que ce soit dans le cadre personnel ou professionnel.

21. Téléphonez-vous régulièrement avec un téléphone sans fil (c'est-à-dire plus d'une fois par semaine) ?

☐ Oui ☐ Non

➥ Si **oui** :

- En quelle année avez-vous commencé à l'utiliser de manière régulière ?
- Pendant combien d'années en tout l'avez-vous utilisé de manière régulière ? ans

➥ Si **non**, passez directement à la question 26, page 10.

*22. Au cours des **3 derniers mois**, combien d'appels par jour en moyenne avez-vous passés ou reçus avec un téléphone sans fil ?*

Aucun	Moins d'un appel par jour	1 à 4 appels par jour	5 à 9 appels par jour	10 à 29 appels par jour	30 appels ou plus par jour
☐	☐	☐	☐	☐	☐
				Soit l'équivalent de 1 à 3 appels par heure, 10 heures par jour	*Soit l'équivalent de 3 appels ou plus par heure, 10 heures par jour*

*23. Au cours des **3 derniers mois**, combien de temps par semaine en moyenne avez-vous passé en communication avec un téléphone sans fil ?*

Aucun	Moins de 5 minutes par semaine	5 à 29 minutes par semaine	30 à 59 minutes par semaine	1 à moins de 4 heures par semaine	4 à moins de 7 heures par semaine	7 heures ou plus par semaine
☐	☐	☐	☐	☐	☐	☐
				Soit l'équivalent de 8 à 34 minutes par jour	*Soit l'équivalent de 35 à 59 minutes par jour*	*Soit l'équivalent de 1 heure ou plus par jour*

*24. Si vous avez actuellement un téléphone sans fil à la maison, où se situe(nt) la (les) base(s) qui sont **branchée(s) sur la prise téléphonique** (prise murale ou prise de votre box internet) ? (plusieurs réponses possibles ; ne comptez pas les bases branchées uniquement sur la prise de courant.)*

☐ Dans la chambre, à moins de 3 mètres de mon lit

☐ Dans la chambre, à plus de 3 mètres de mon lit

☐ Dans une autre pièce

☐ Je n'ai pas ce type de base à la maison

III. VOTRE UTILISATION DE DISPOSITIFS D'ACCES SANS FIL A INTERNET, AUTRES QUE LE TELEPHONE MOBILE

*25. Au cours des **3 derniers mois**, vous êtes-vous connecté(e) à Internet pendant au moins une heure par semaine avec n'importe lequel des dispositifs d'**accès sans fil** listés ci-dessous ? (N'incluez pas ici l'accès sans fil à Internet en utilisant un (des) téléphone(s) mobile(s).)*

- Ordinateur fixe, ordinateur portable, tablette (par exemple iPad, Samsung Galaxy Tab),
- Liseuse de livres électroniques (par exemple Kindle, Kobo),
- Baladeur musical numérique (par exemple iPod, Sony Z-Series Walkman),
- Console portable de jeux vidéo (par exemple Sony PSP, Nintendo DS).

☐ Oui ☐ Non

➥ Si **non**, passez directement à la question 28, page 11.

*26. Au cours des **3 derniers mois**, combien d'heures par jour en moyenne avez-vous utilisé l'accès **sans fil** à Internet de chacun des dispositifs ci-dessous ?*

- Pour un jour de <u>semaine « typique »</u>

	Je n'utilise pas ce dispositif	Aucune ou presque	Moins d'1 heure par jour	1 à moins de 4 heures par jour	4 à moins de 7 heures par jour	7 heures ou plus par jour
Ordinateur fixe	☐	☐	☐	☐	☐	☐
Ordinateur portable	☐	☐	☐	☐	☐	☐
Tablette (par ex. iPad, Samsung Galaxy Tab)	☐	☐	☐	☐	☐	☐
Liseuse de livres électroniques (par ex. Kindle, Kobo)	☐	☐	☐	☐	☐	☐
Baladeur numérique, lecteur mp3/mp4 (par ex. iPod, Sony Z-Series Walkman)	☐	☐	☐	☐	☐	☐
Console portable de jeux vidéo (par ex. Sony PSP, Nintendo DS)	☐	☐	☐	☐	☐	☐

- Pour un jour de <u>weekend « typique »</u>

	Je n'utilise pas ce dispositif	Aucune ou presque	Moins d'1 heure par jour	1 à moins de 4 heures par jour	4 à moins de 7 heures par jour	7 heures ou plus par jour
Ordinateur fixe	☐	☐	☐	☐	☐	☐
Ordinateur portable	☐	☐	☐	☐	☐	☐
Tablette (par ex. iPad, Samsung Galaxy Tab)	☐	☐	☐	☐	☐	☐
Liseuse de livres électroniques (par ex. Kindle, Kobo)	☐	☐	☐	☐	☐	☐

Baladeur numérique, lecteur mp3/mp4 (par ex. iPod, Sony Z-Series Walkman)	☐	☐	☐	☐	☐	☐
Console portable de jeux vidéo (par ex. Sony PSP, Nintendo DS)	☐	☐	☐	☐	☐	☐

Supplementary Figure S1: Distribution of Education Duration: Stacked Percentages of Individuals by Years of Education within Each Age and Sex Category, COSMOS-France Cohort, 2017-19 (N=18,231).

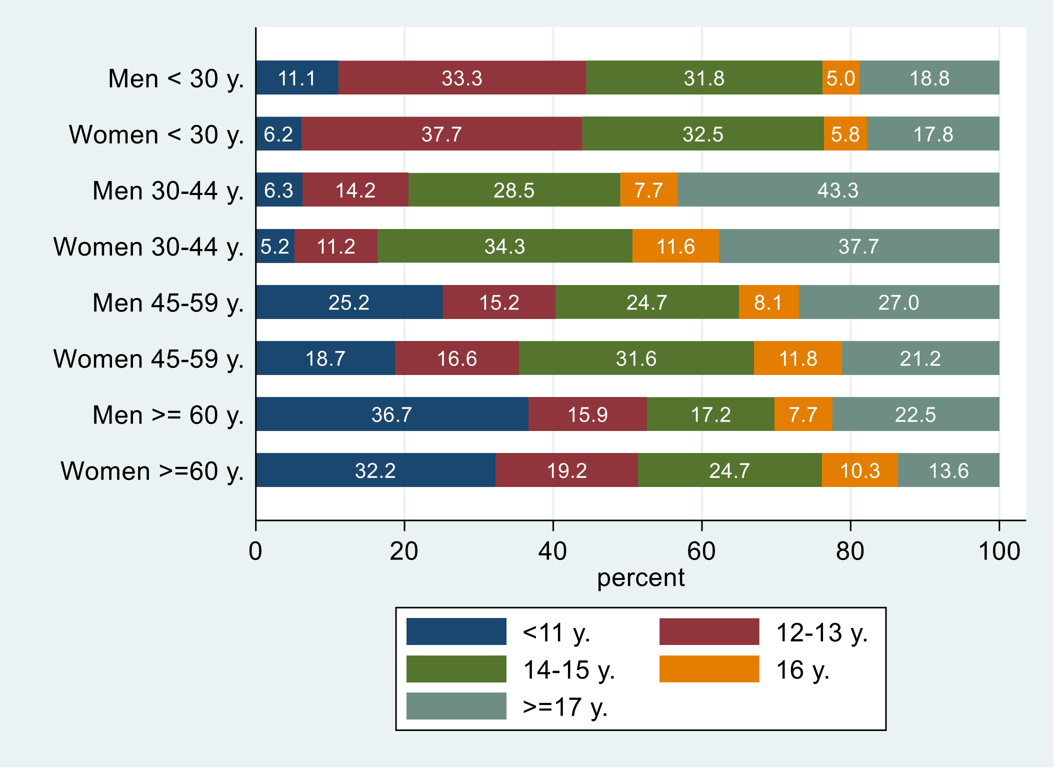

Notes: y. means years. Percentages are shown excluding missing values, presented in white font. Generalized ordered logistic regression, modeling education duration categories as a function of age categories, produced trend test p-values for age of < 0.005 across all thresholds.

Supplementary Figure S2: Distribution of Smoking Status: Stacked Percentages of Never Smokers, Ex-Smokers, and Smokers within Each Age and Sex Category, COSMOS-France Cohort, 2017-2019 (N=17,755).

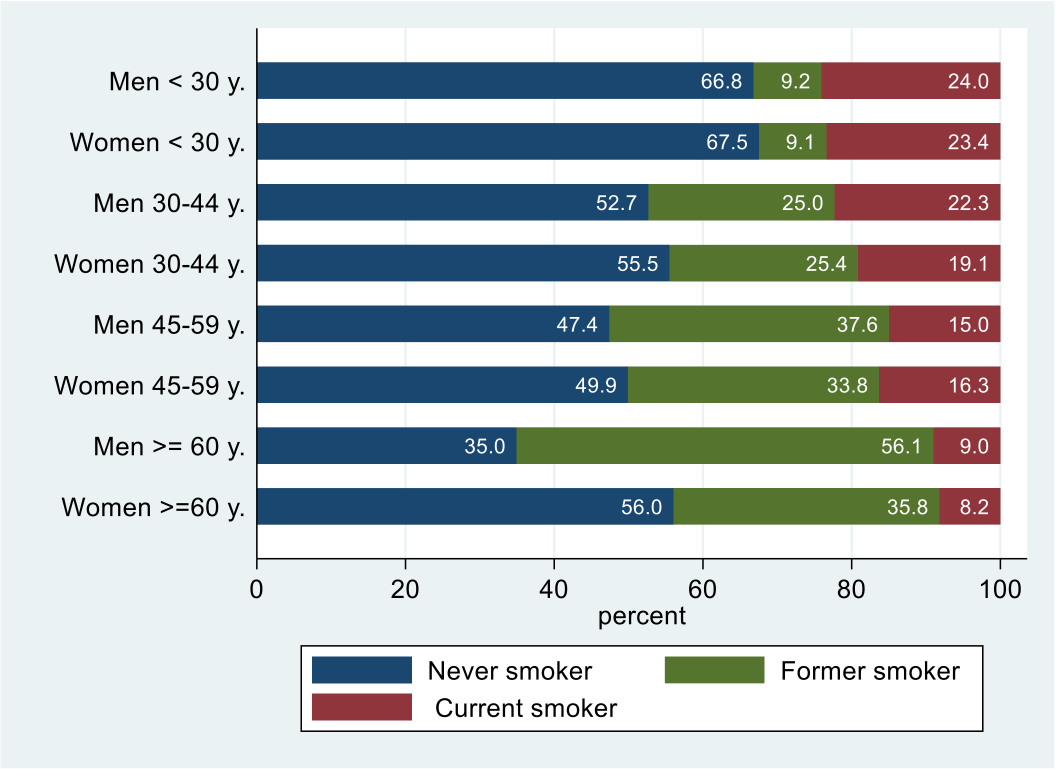

Notes: y. means years. Percentages are shown excluding missing values, presented in white font. Logistic regression, modeling ever versus never smoking as a function of age categories and sex, produced trend test p-values for age of < 0.005, and p-value for sex <0.005; logistic regression modelling ex-smokers versus current smokers as a function of age categories and sex, produced trend test p-values for age of < 0.005 and p-value for sex <0.005.

Supplementary Figure S3: Distribution of Body Mass Index (BMI): Stacked Percentages of Underweight, Healthy Weight, Overweight, and Obese Individuals within Each Age and Sex Category, COSMOS-France Cohort, 2017-19 (N=18,218).

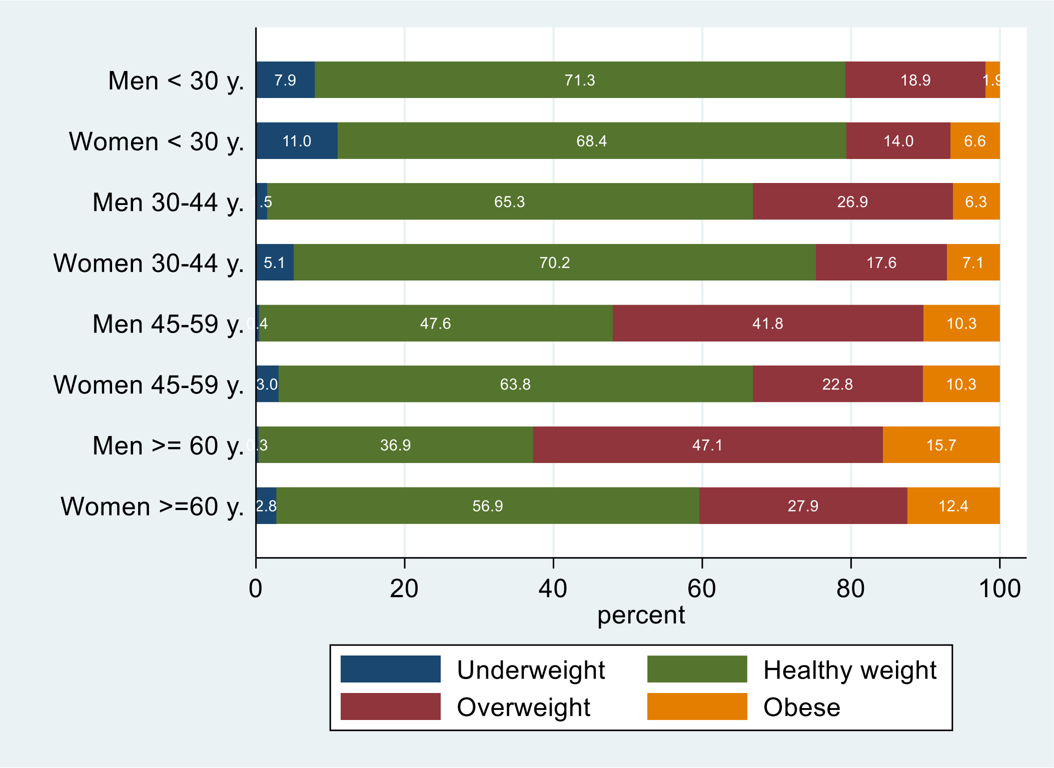

Notes: y. means years. Percentages are shown excluding missing values, presented in white font. Generalized ordered logistic regression, modeling BMI categories as a function of age categories, produced trend test p-values for age of < 0.005 across all thresholds.

Supplementary Figure S4: Distribution of Handedness: Stacked Percentages of Left-Handed, Ambidextrous and Right-Handed Individuals within Each Age and Sex Category, COSMOS-France Cohort, 2017-19 (N=18,296).

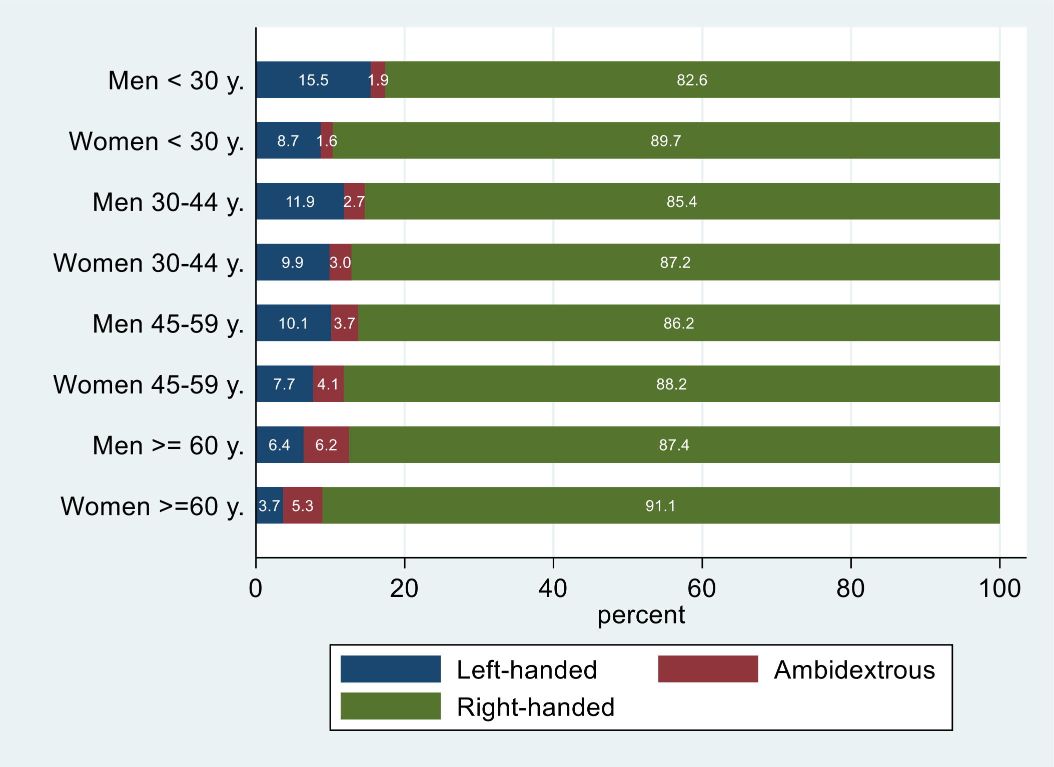

Notes: y. means years. Percentages are shown excluding missing values, presented in white font. Multinomial logistic regression modeling handedness as a function of age categories and sex, produced trend test p-values for age of < 0.005, and p-value for sex <0.005. Generalized ordered logistic regression modeling education or BMI categories as a function of handedness after adjustment for 8 cross-categorisation of age and sex did not show significant associations, but logistic regression of ever versus never smoking showed that left-handed individuals comprised a higher proportion of smokers and ex-smokers compared to right-handed individuals (Odds Ratio=1.15, 95% Confidence Interval 1.02-1.28).

Supplementary Figure S5: Proportion of self-reported duration of mobile phone use by age group for a) men and b) women in the COSMOS-France Cohort, 2017-19.

A) Men

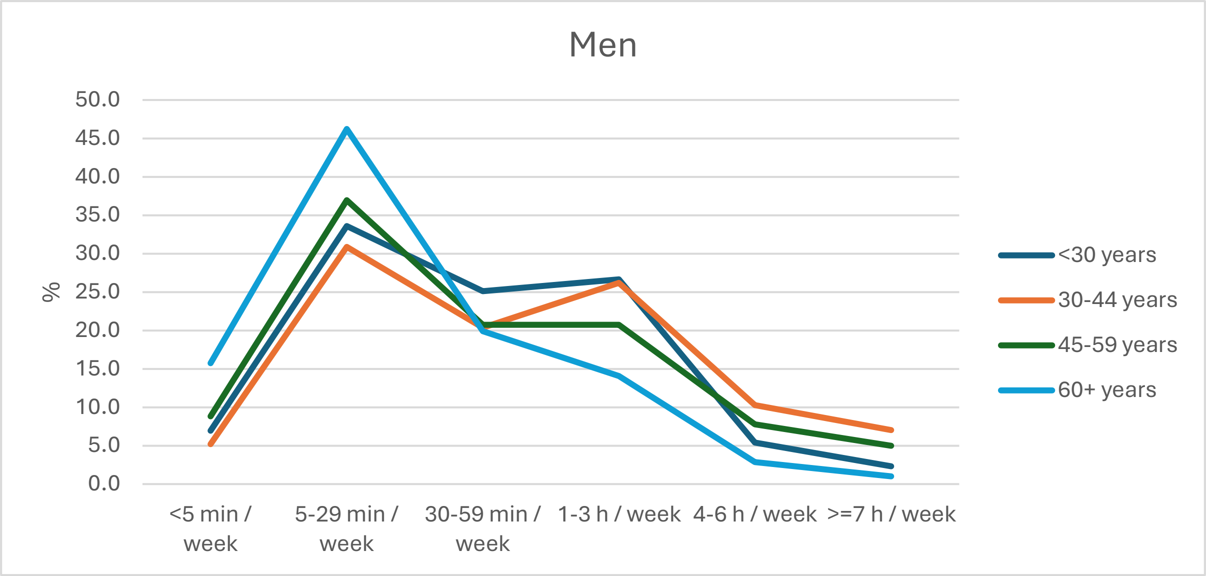

B) Women

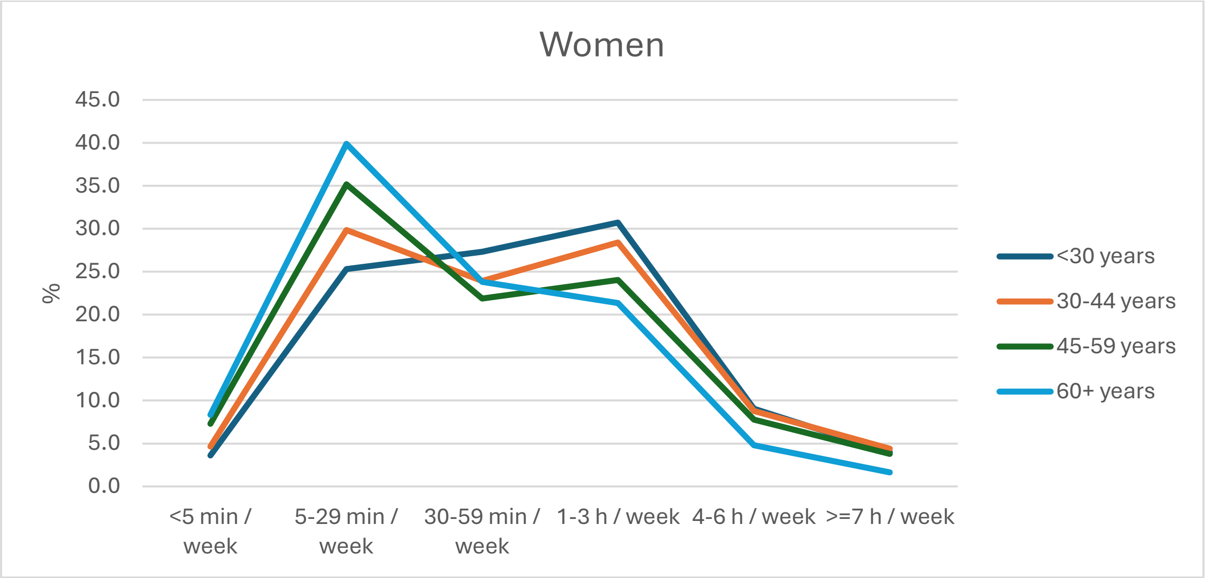

Supplementary Figure S6: Distribution of Laterality of Mobile Phone Use by Handedness: Stacked Percentages of Left-Side, Equal-Side, Right-Side Mobile Phone Users, and Users Who Never Hold Their Phone Close to Their Head within Each Age and Sex Category among Left-Handed and Ambidextrous and among Right -Handed Participants COSMOS-France cohort, 2017-2019 (N=18,296).

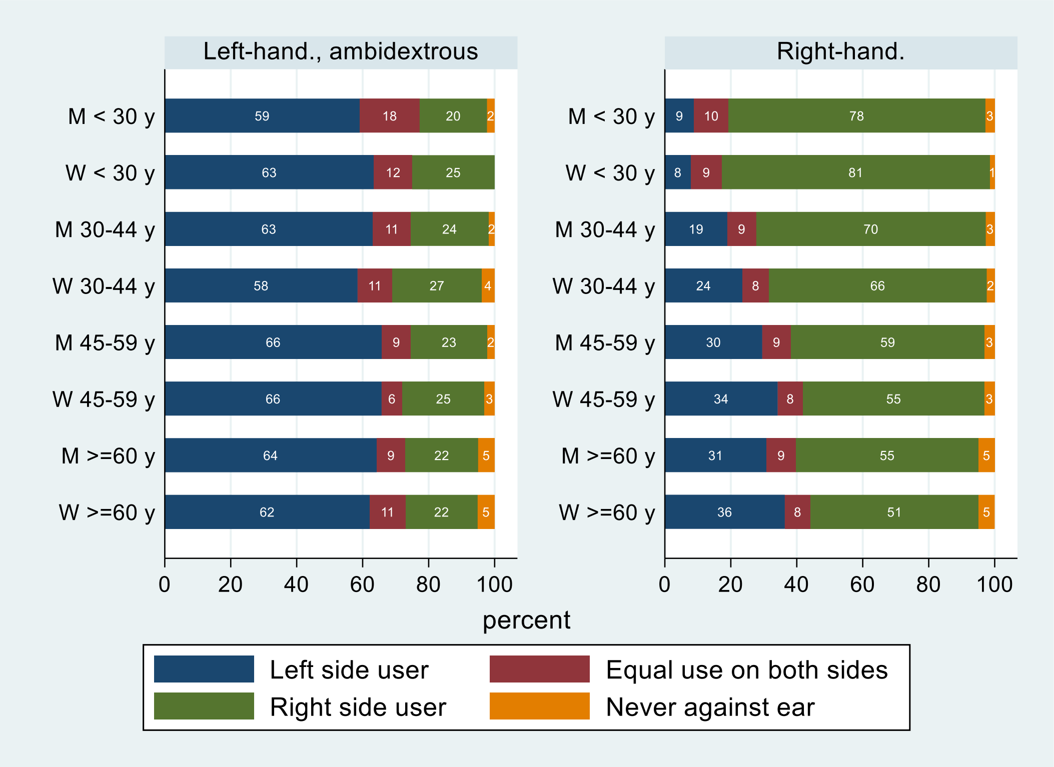

Notes: M stands for Men, W stands for Women, y means years and hand. stands for handed. Percentages are shown excluding missing values and rounded for clarity, presented in white font.

Supplementary Figure S7: Distribution of Weekly Mobile Phone Usage (stacked proportion of each category) over Participants' Lifetime Usage History, by Period of First Use, for a) men and b) women, COSMOS-France cohort, 2017-19.

A) Men

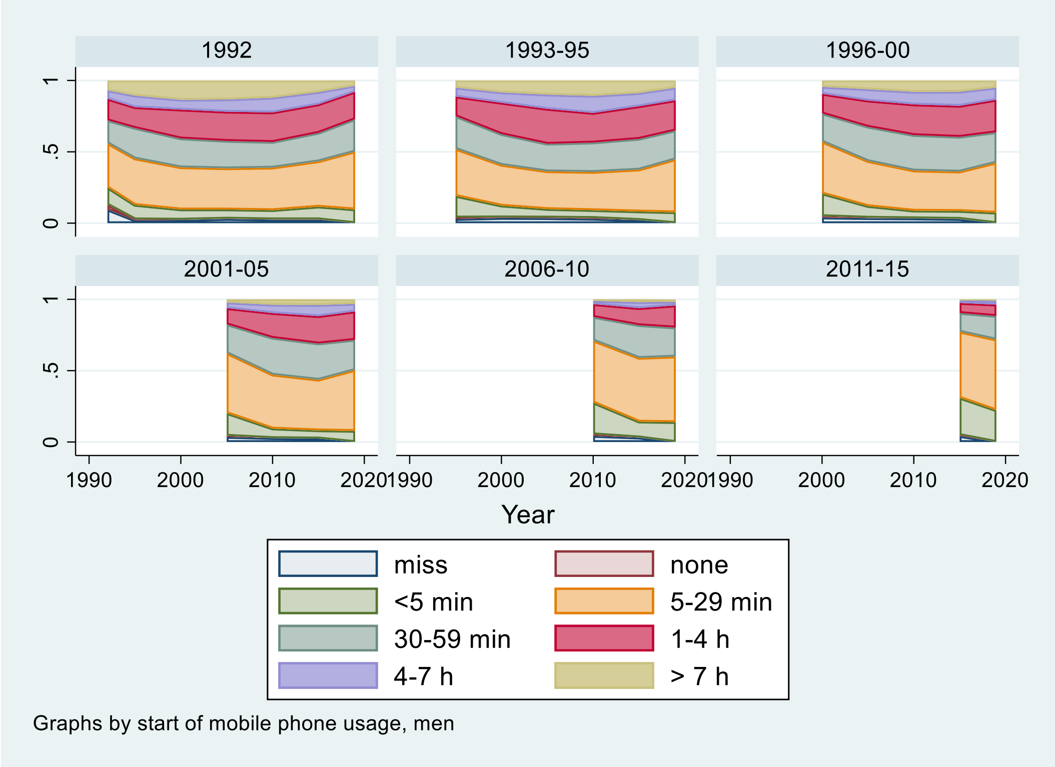

B) Women

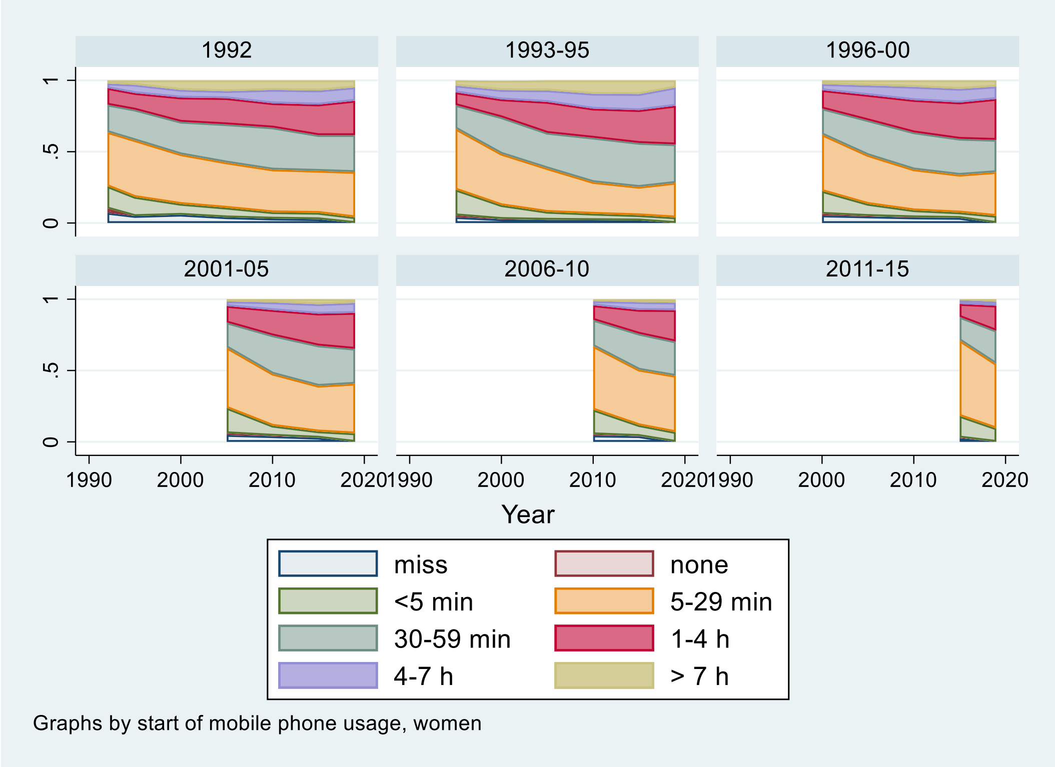

Notes: min means minutes, h means hour.

Supplementary Figure S8: Distribution Cordless Phone (DECT) Usage and Duration: Stacked Percentages of No Use and Duration of Cordless Phone Usage within Categories of Mobile Phone Use Duration, COSMOS-France Cohort, 2017-19 (N= 17,351).

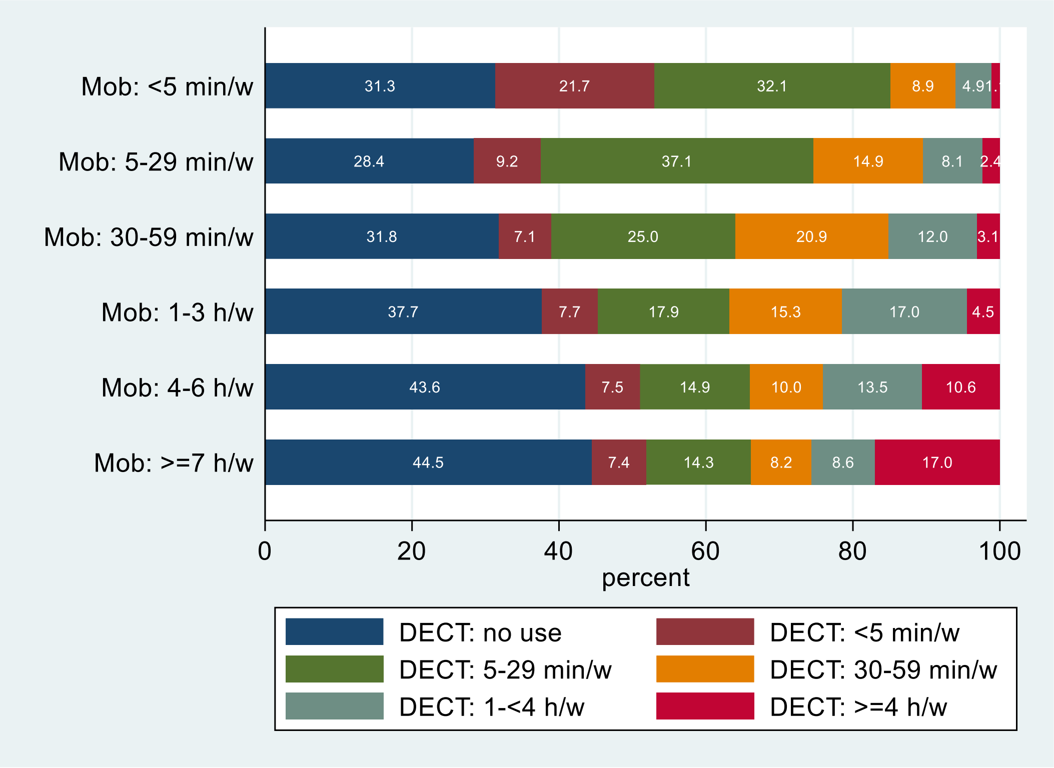

Notes: min means minutes, h means hour, w means week, Mob means mobile phone, DECT means cordless phone. Percentages are shown excluding missing values and rounded for clarity, presented in white font.

Supplementary Table S1: Overall participation by age and sex in the COSMOS-France study, 2017-19

Characteristics	Participants in the COSMOS-France study* N	[%]	*Participants who consented to provision of operator data+* *N*	*(%)$*	Non-participants N	[%]
**Total**	**19,013**	**[48.4]**	*15,856*	*(83.4)*	**20,271**	**[51.6]**
**Sex and age**						
Men < 30 years	274	[1.4]	*243*	*(88.7)*	436	[2.1]
Women < 30 years	529	[2.8]	*458*	*(86.6)*	781	[3.8]
Men 30-44 years	1,672	[8.8]	*1,451*	*(86.8)*	2,101	[10.4]
Women 30-44 years	2,401	[12.6]	*2,139*	*(89.1)*	2,563	[12.6]
Men 45-59 years	2,942	[15.5]	*2,470*	*(84.0)*	3,201	[15.8]
Women 45-59 years	3,582	[18.8]	*2,992*	*(83.5)*	3,881	[19.1]
Men 60 years and above	3,589	[18.9]	*2,924*	*(81.5)*	3,354	[16.5]
Women 60 years and above	4,024	[21.2]	*3,179*	*(79.0)*	3,954	[19.5]

*Notes: *Logistic regression, modeling participation as a function of sex and age categories, produced a trend test p-value for age of < 0.005 and a p-value for sex that was not significant (ns).*

+ Logistic regression, modeling consent to operator data transfer as a function of sex and age categories, produced a trend test p-value for age of < 0.005 and a p-value for sex that was not significant (ns). $ Percentage computed as those consenting to provision of operator data out of the participants.

Supplementary Table S2: Odds ratio (OR) and 95% confidence interval (CI) of no use of mobile phone associated with education, smoking, body mass index, and handedness, COSMOS France, 2017-19.

	N+	OR for no use*	95 % CI	OR for no use**	95 % CI
**Education duration (years)**					
≤11	238	1.53	(1.23 ; 1.89)		
12-13	122	1.21	(0.95 ; 1.55)		
14-15	150	1.00	reference		
16	49	0.86	(0.62 ; 1.20)		
17 or more	124	0.89	(0.70 ; 1.14)		
trend test p-value		*<0.01*			
**Smoking status**					
Never smoker	372	1.00	reference	1.00	reference
Former smoker	237	0.74	(0.62 ; 0.88)	0.73	(0.62 ; 0.87)
Smoker	61	0.62	(0.47 ; 0.82)	0.62	(0.47 ; 0.81)
trend test p-value		*<0.01*		*<0.01*	
**Body mass index**					
Underweight	16	1.21	(0.72 ; 2.02)	1.26	(0.75 ; 2.10)
Healthy weight	342	1.00	reference	1.00	reference
Overweight	226	1.03	(0.86 ; 1.23)	0.98	(0.82 ; 1.18)
Obese	96	1.25	(0.99 ; 1.58)	1.17	(0.92 ; 1.48)
trend test p-value		*0.18*		*0.49*	
**Handedness**					
Right-handed	587	1.00	reference	1.00	reference
Left-handed or ambidextrous	88	1.13	(0.90 ; 1.42)	1.14	(0.91 ; 1.44)

+ number of non-mobile phone users in category

**logistic regression models of no use versus use of mobile phone, adjusted for 8 categories of age and sex*

***logistic regression models of no use versus use of mobile phone, adjusted for 8 categories of age and sex and for education*

Supplementary Table S3: Predicted probabilities of duration of use, for mobile phone users, according to education length, smoking status, body mass index and handedness, COSMOS France, 2017-19.

	Duration of mobile phone use					
	<5 min / week	5-29 min / week	30-59 min / week	1-3 h / week	4-6 h / week	>=7 h / week
**Education duration⁺ (years)**			N=17,507			
≤11	0.12	0.43	0.21	0.18	0.05	0.02
12-13	0.09	0.39	0.22	0.21	0.06	0.03
14-15	0.08	0.36	0.23	0.23	0.07	0.03
16	0.07	0.35	0.23	0.24	0.07	0.04
17 or more	0.06	0.32	0.23	0.26	0.08	0.04
trend test p-value						*<0.01*
**Smoking status***			N=17,048			
Never smoker	0.09	0.38	0.22	0.21	0.06	0.03
Former smoker	0.08	0.36	0.22	0.23	0.07	0.04
Smoker	0.08	0.35	0.22	0.24	0.07	0.04
trend test p-value						*<0.01*
**Body mass index***			N=17,237			
Underweight	0.11	0.41	0.21	0.20	0.05	0.03
Healthy weight	0.09	0.38	0.22	0.22	0.06	0.03
Overweight	0.08	0.36	0.22	0.23	0.07	0.04
Obese	0.08	0.36	0.22	0.23	0.07	0.04
*p-value***						*0.99*
**Handedness***			N=17,332			
Right-handed	0.09	0.37	0.22	0.22	0.07	0.03
Left-handed or ambidextrous	0.08	0.38	0.20	0.23	0.07	0.04
*p-value***						*0.19*

Note: ⁺predicted probabilities from generalised ordered logistic models adjusted for age and sex (8 cross-categories).

** predicted probabilities from generalised ordered logistic models adjusted for age and sex (8 cross-categories) and education.*

***likelihood ratio test of model with variable vs without*

Supplementary Table S4: Description of frequency of calls among mobile phone users in 2017-19, COSMOS-France study

	Age groups among men								Age groups among women								Total	
	<30 years		30-44 years		45-59 years		60+ years		<30 years		30-44 years		45-59 years		60+ years		Total	
	N	%	N	%	N	%	N	%	N	%	N	%	N	%	N	%	N	%
**Current mobile phone users**	259	100.0	1577	100.0	2765	100.0	3279	100.0	498	100.0	2275	100.0	3406	100.0	3723	100.0	17782	100.0
**Frequency of voice calls (calls/day, among current mobile phone users)**																		
<1	92	35.5	324	20.6	530	19.2	956	29.2	137	27.5	559	24.6	900	26.4	917	24.6	4415	24.8
1-4	127	49.0	743	47.1	1278	46.2	1716	52.3	288	57.8	1273	56.0	1785	52.4	2198	59.0	9408	52.9
5-9	28	10.8	333	21.1	637	23.0	502	15.3	60	12.1	341	15.0	583	17.1	546	14.7	3030	17.0
>=10	12	4.6	177	11.2	319	11.5	104	3.2	13	2.6	101	4.4	138	4.1	60	1.6	924	5.2
missing	0	0.0	0	0.0	1	0.0	1	0.0	0	0.0	1	0.0	0	0.0	2	0.1	5	0.0

Supplementary Table S5: Effect of education, smoking status, body mass index and handedness on year of start of mobile phone use among mobile phone users, COSMOS-France study, 2017-2019

	N*	coefficient*	95 % CI	coefficient**	95 % CI
**Education duration (years)**					
≤11	3666	0.63	(0.38 ; 0.88)	0.69	(0.43 ; 0.95)
12-13	2832	0.20	(-0.07 ; 0.46)	0.26	(-0.01 ; 0.53)
14-15	4549	0.00	reference	0.00	reference
16	1650	-0.10	(-0.42 ; 0.22)	-0.18	(-0.51 ; 0.15)
17 or more	4297	-0.26	(-0.50 ;- 0.02)	-0.40	(-0.64 ;- 0.16)
**Smoking status***					
Never smoker	8149	0.0	reference	0.00	reference
Former smoker	5991	-0.97	(-1.16 ;- 0.78)	-0.95	(-1.15 ;- 0.76)
Current smoker	2405	-1.14	(-1.40 ;- 0.88)	-1.20	(-1.46 ;- 0.94)
**Body mass index***					
Underweight	425	0.64	(0.09 ; 1.19)	0.61	(0.05 ; 1.17)
Healthy weight	9574	0.0	reference	0.0	reference
Overweight	5203	-0.50	(-0.70 ;- 0.31)	-0.55	(-0.75 ;- 0.35)
Obese	1776	-0.86	(-1.15 ;- 0.57)	-0.98	(-1.27 ;- 0.68)
**Handedness***					
Right-handed	15,011	0.0	reference	0.0	reference
Left-handed or ambidextrous	2,057	0.12	(-0.14 ; 0.38)	0.18	(-0.09 ; 0.45)

**linear regression models of year of start of use adjusted for 8 categories of age and sex. Numbers of participants reported correspond to numbers included in regression.*

***linear regression models of year of start of use, adjusted for 8 categories of age and sex, for education, smoking status and body mass index. Two models were adjusted, one without handedness variable (results reported in the first lines) and one with handedness variable (results for handedness coefficients reported in handedness panel).*
